# Bioenergetic impairment in schizophrenia: role of mitochondrial signaling in synaptic dysfunction - a systematic review

**DOI:** 10.3389/fcell.2026.1740079

**Published:** 2026-02-27

**Authors:** Valerio Ricci, Giovanni Martinotti, Alessio Mosca, Giuseppe Maina

**Affiliations:** 1 University of Turin, San Luigi Gonzaga Hospital, Turin, Italy; 2 Department of Neurosciences, Imaging and Clinical Sciences, Università degli Studi G. D’Annunzio Chieti-Pescara, Chieti, Italy; 3 Department of Neurosciences “Rita Levi Montalcini”, University of Turin, Turin, Italy

**Keywords:** bioenergetics, first-episode psychosis, mitochondrial dysfunction, oxidative phosphorylation, schizophrenia, synaptic dysfunction

## Abstract

**Background:**

Mitochondrial dysfunction represents a critical pathophysiological mechanism in schizophrenia, potentially linking bioenergetic impairment to synaptic dysfunction and cognitive deficits. Converging evidence suggests that deficits in oxidative phosphorylation may drive the synaptic pathology contributing to treatment-resistant cognitive and negative symptoms.

**Objective:**

To systematically review the evidence linking mitochondrial bioenergetic dysfunction to synaptic impairment in schizophrenia, examining structural, functional, and molecular mechanisms across multiple methodological approaches.

**Methods:**

Following PRISMA guidelines, we searched PubMed/MEDLINE, Embase, PsycINFO, and Web of Science from 2000 to 2025 for original research studies investigating mitochondrial function and synaptic dysfunction in schizophrenia. Two independent reviewers screened 2,224 articles, with 29 studies meeting inclusion criteria. Quality was assessed using the Newcastle-Ottawa Scale (median score 7/9).

**Results:**

Twenty-nine studies representing 2,847 participants demonstrated consistent mitochondrial dysfunction across *postmortem* (n = 10), neuroimaging (n = 8), and molecular/cellular (n = 11) investigations. *Postmortem* studies revealed reduced complex I (18%–35%) and complex IV activity (22%–28%) in prefrontal cortex, with concurrent synaptic density reductions (27%). Neuroimaging studies demonstrated 20%–22% reductions in ATP synthesis rates correlating with cognitive deficits (r = 0.48) and negative symptoms (r = −0.42). First-episode antipsychotic-naïve patients exhibited comparable bioenergetic abnormalities, indicating primary pathophysiology rather than medication effects. Molecular studies identified impaired calcium homeostasis, oxidative stress (27%–35% glutathione reductions in synaptic compartments), and novel pseudogene regulatory mechanisms perpetuating complex I deficits. Peripheral biomarkers including platelet complex I activity and cell-free mitochondrial DNA showed disease specificity and correlation with cognitive impairment. Substantial methodological heterogeneity precluded meta-analysis but provided complementary evidence across analytical levels.

**Conclusion:**

Mitochondrial bioenergetic impairment represents a core, potentially modifiable pathophysiological mechanism driving synaptic dysfunction in schizophrenia. Regional specificity (prefrontal cortex, hippocampus) and cell-type selectivity (pyramidal neurons) provide mechanistic insights into cognitive symptom profiles. Early presence and progressive worsening suggest critical intervention windows. Mitochondrial-targeted therapies merit investigation as novel approaches for treatment-resistant cognitive and negative symptoms.

## Introduction

Schizophrenia represents one of the most debilitating psychiatric disorders, affecting approximately 1% of the global population and characterized by profound disturbances in cognition, perception, and social functioning (McGrath et al., 2008; Charlson et al., 2018). Despite decades of intensive research, the fundamental pathophysiological mechanisms underlying schizophrenia remain incompletely understood (Insel, 2010; Owen et al., 2016). Recent advances in neuroscience have increasingly implicated mitochondrial dysfunction as a central feature of schizophrenia pathology, potentially representing a convergent mechanism linking genetic susceptibility, environmental risk factors, and clinical manifestations (Prabakaran et al., 2004; Clay et al., 2011; Rajasekaran et al., 2015).

The dopamine hypothesis has long provided the predominant framework for understanding schizophrenia, particularly positive symptoms. Evidence from amphetamine-induced psychosis and the efficacy of dopamine D2 receptor antagonists in treating hallucinations and delusions established striatal dopamine hyperactivity as a core pathophysiological mechanism (McCutcheon et al., 2020; Buck et al., 2022). However, dopaminergic dysfunction alone cannot fully explain the cognitive deficits and negative symptoms that often dominate the clinical picture and predict functional outcomes (Keefe, 2007). Emerging evidence suggests that mitochondrial bioenergetic dysfunction may represent an upstream, convergent mechanism affecting multiple neurotransmitter systems, including dopaminergic pathways, and thereby contributing to the full symptom spectrum of the disorder.

The conceptualization of schizophrenia as a disorder of synaptic connectivity has gained substantial empirical support over the past 2 decades (Stephan et al., 2009). Structural neuroimaging studies have consistently demonstrated reductions in gray matter volume, particularly in prefrontal and temporal regions (Honea et al., 2005), while *postmortem* investigations have revealed decreased dendritic spine density on pyramidal neurons, especially in cortical layer III (Glantz and Lewis, 2000; Garey et al., 1998; Kolluri et al., 2005). These synaptic abnormalities correlate with cognitive deficits and negative symptoms that, alongside positive symptoms, constitute the core clinical manifestations of schizophrenia (Green et al., 2000; Milev et al., 2005), suggesting that impaired synaptic function contributes to the core pathological manifestations of the disorder.

Synaptic neurotransmission is among the most energy-demanding processes in the brain, consuming approximately 80%–90% of cerebral ATP (Attwell and Laughlin, 2001; Harris et al., 2012). The maintenance of ionic gradients essential for action potential generation and propagation, vesicular neurotransmitter cycling, and receptor trafficking all require substantial and sustained energy supply (Rangaraju et al., 2014; Pathak et al., 2015). This high energetic demand renders synapses particularly vulnerable to bioenergetic dysfunction, establishing a critical link between mitochondrial impairment and synaptic pathology (Cheng et al., 2010; Li et al., 2004). Mitochondria serve as the primary energy-generating organelles in neurons, producing over 95% of cellular ATP through oxidative phosphorylation (OXPHOS) (Kann and Kovács, 2007; Yadava and Nicholls, 2007; Xuan et al., 2015). The electron transport chain (ETC), comprising complexes I through V located on the inner mitochondrial membrane, transfers electrons derived from NADH and FADH_2_ to molecular oxygen, establishing a proton gradient that drives ATP synthesis (Mitchell, 1961; Saraste, 1999). This process is remarkably efficient under normal conditions, generating approximately 30–32 ATP molecules per glucose molecule (Rich, 2003). Beyond their bioenergetic role, mitochondria are critical regulators of calcium homeostasis, reactive oxygen species (ROS) production, and apoptotic signaling—all processes implicated in schizophrenia pathophysiology (Duchen, 2000; Brookes et al., 2004; Green and Reed, 1998). Mitochondria buffer cytosolic calcium through the mitochondrial calcium uniporter (MCU), preventing excessive calcium accumulation that could trigger excitotoxicity (Baughman et al., 2011; De Stefani et al., 2011). However, this buffering capacity is dependent on adequate mitochondrial membrane potential and ATP availability, creating a reciprocal relationship between energy status and calcium handling (Nicholls, 2005).

Neuronal mitochondria exhibit specialized properties adapted to the unique demands of synaptic transmission (Hollenbeck and Saxton, 2005; MacAskill and Kittler, 2010). Synaptic mitochondria are strategically positioned at presynaptic terminals and dendritic spines, where they buffer calcium transients associated with neurotransmitter release and postsynaptic depolarization (Billups and Forsythe, 2002; Levy et al., 2003). Studies using fluorescence microscopy have demonstrated that mitochondria actively transport along axons and dendrites, accumulating at synapses with high activity levels (Chang et al., 2006; Saotome et al., 2008). This dynamic positioning allows mitochondria to meet local energy demands and regulate synaptic calcium dynamics with spatial and temporal precision (Sheng and Cai, 2012). Early observations of metabolic abnormalities in schizophrenia date back over a century (Horrobin, 1977). However, systematic investigation of mitochondrial function in schizophrenia began in earnest in the 1990s with the advent of molecular and neuroimaging techniques capable of assessing brain energy metabolism *in vivo* (Buchsbaum et al., 1982; Weinberger et al., 1986). Positron emission tomography (PET) studies revealed hypofrontality—reduced glucose metabolism in prefrontal regions—during cognitive tasks in schizophrenia patients, suggesting impaired energy utilization in cortical networks critical for executive function (Andreasen et al., 1992; Carter et al., 1998).

Subsequent *postmortem* investigations provided direct molecular evidence for mitochondrial dysfunction, demonstrating alterations in ETC complex expression and activity, mitochondrial DNA (mtDNA) abnormalities, and ultrastructural changes in mitochondrial morphology (Maurer et al., 2001; Altar et al., 2005; Whatley et al., 1996). Gene expression studies identified downregulation of nuclear-encoded mitochondrial genes in schizophrenia brain tissue, with transcriptomic analyses revealing coordinated dysregulation of OXPHOS pathways (Middleton et al., 2002; Iwamoto et al., 2005). More recently, magnetic resonance spectroscopy (MRS) studies have enabled non-invasive measurement of high-energy phosphate metabolism in living patients, revealing consistent abnormalities in ATP synthesis and phosphocreatine metabolism (Keshavan et al., 2000; Keshavan et al., 2002).

These converging lines of evidence across multiple methodological approaches have established mitochondrial dysfunction as a robust finding in schizophrenia research (Ben-Shachar and Laifenfeld, 2004; Martins-de-Souza et al., 2009). However, the specific mechanisms linking mitochondrial impairment to synaptic pathology—and ultimately to clinical symptoms—require systematic integration and analysis. Despite accumulating evidence for both mitochondrial dysfunction and synaptic pathology in schizophrenia, several critical questions remain unanswered. First, how does mitochondrial bioenergetic impairment specifically affect synaptic function at the molecular, cellular, and circuit levels? Second, are certain brain regions or synaptic populations selectively vulnerable to mitochondrial dysfunction? Third, what are the temporal dynamics of mitochondrial-synaptic dysfunction during illness progression? Fourth, how do mitochondrial abnormalities relate to specific symptom domains—positive, negative, and cognitive symptoms (Addington et al., 2012; Keefe and Harvey, 2012)?

Previous reviews have examined mitochondrial abnormalities or synaptic deficits as separate topics (Nucifora et al., 2017; Steullet et al., 2016), but systematic integration of these findings is lacking. Furthermore, the regional specificity of mitochondrial-synaptic dysfunction, relationships to clinical symptomatology, and potential for therapeutic intervention require comprehensive synthesis.

This systematic review addresses these gaps by: (1) synthesizing evidence from *postmortem*, neuroimaging, and molecular studies linking mitochondrial dysfunction to synaptic impairment in schizophrenia; (2) examining regional and cell-type specific patterns of mitochondrial-synaptic pathology; (3) elucidating mechanisms connecting bioenergetic deficits to specific synaptic abnormalities; (4) identifying associations between mitochondrial dysfunction and clinical symptom domains; and (5) discussing implications for novel therapeutic approaches targeting mitochondrial function.

## Methods

### Study design and registration

**FIGURE 1:**
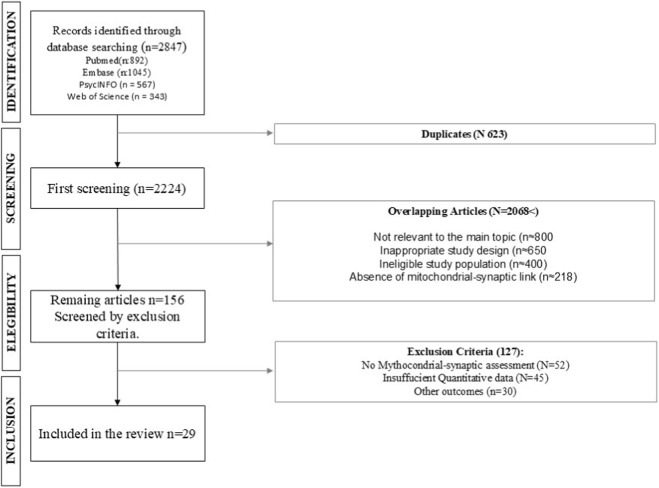
PRISMA flow diagram of the study selection process.

This systematic review was conducted according to the Preferred Reporting Items for Systematic Reviews and Meta-Analyses (PRISMA) 2020 guidelines (Page et al., 2021) (Figure 1; Supplementary Table A). The review protocol was prospectively registered with PROSPERO (registration CRD420251184449, November 2025).

### Search strategy

A comprehensive literature search was conducted in four major electronic databases: PubMed/MEDLINE, Embase, PsycINFO, and Web of Science. The search covered publications from January 2000 to October 2025, focusing on the modern era of molecular and neuroimaging research. The search strategy combined four concept domains using Boolean operators (Supplementary Tables A and B):

- Mitochondrial terms: “mitochondria” OR “mitochondrial dysfunction” OR “electron transport chain” OR “oxidative phosphorylation” OR “OXPHOS” OR “complex I” OR “complex IV” OR “ATP” OR “bioenergetics” OR “energy metabolism” OR “creatine kinase” OR “mtDNA”
- Synaptic terms: “synapse” OR “synaptic” OR “neurotransmission” OR “synaptic plasticity” OR “dendritic spine” OR “presynaptic” OR “postsynaptic” OR “vesicle release” OR “neurotransmitter” OR “synaptic density”
- Calcium/ROS terms: “calcium homeostasis” OR “calcium buffering” OR “calcium dysregulation” OR “oxidative stress” OR “reactive oxygen species” OR “ROS” OR “glutathione” OR “antioxidant”
- Disorder terms: “schizophrenia” OR “psychosis” OR “psychotic” OR “first-episode psychosis” OR “schizophrenia spectrum” OR “early psychosis”

Additional searches included manual review of reference lists from included articles and forward citation tracking using Google Scholar and Web of Science. No language restrictions were applied initially, though non-English articles were translated when necessary.

This search strategy was designed to specifically examine the mitochondrial-synaptic interface in schizophrenia. While dopaminergic system terms were not explicitly included in our search string, our focus was to comprehensively synthesize evidence linking bioenergetic dysfunction to synaptic pathology—a mechanistic pathway that has received less systematic attention despite its potential relevance to treatment-resistant symptoms. This focused approach enabled in-depth examination of this specific pathway while acknowledging that other neurotransmitter systems, including dopaminergic pathways, play crucial roles in schizophrenia pathophysiology.

### Eligibility criteria

Inclusion Criteria: Original research articles published in peer-reviewed journals; Studies including participants diagnosed with schizophrenia, schizophrenia spectrum disorders, or first-episode psychosis according to DSM or ICD criteria; Studies examining mitochondrial function using *postmortem* tissue analysis, peripheral measurements, neuroimaging, or animal models; Studies assessing synaptic outcomes including synaptic density, neurotransmitter function, synaptic plasticity, or electrophysiological measures; Quantitative analysis linking mitochondrial parameters to synaptic outcomes.

Exclusion Criteria: Case reports, case series, conference abstracts, review articles, meta-analyses, or systematic reviews; Studies focusing exclusively on other psychiatric disorders without schizophrenia patients; Studies examining only peripheral mitochondrial measures without brain-relevant outcomes; Animal studies without clear relevance to schizophrenia pathophysiology; Studies without quantitative data suitable for synthesis (Supplementary Tables B and C)

### Study selection

Two independent reviewers (V.R. and G.M.) screened titles and abstracts of all identified records using predefined eligibility criteria. Full-text articles were then independently reviewed by the same reviewers. Disagreements at both stages were resolved through discussion, with a third reviewer (G.Ma.) consulted when consensus could not be reached. Inter-rater reliability was calculated using Cohen’s kappa coefficient.

### Data extraction

Data extraction was performed independently by two reviewers using a standardized form. The following information was extracted: study characteristics (author, year, country, design, sample size); participant demographics (age, gender, diagnosis, illness duration, medication status); mitochondrial measures (ETC complex activity, ATP levels, calcium handling, oxidative stress markers); synaptic outcomes (density, morphology, function); neuroimaging parameters (scanner specifications, analysis methods); statistical analyses (effect sizes, correlations, p-values); and study quality indicators.

### Quality assessment

Methodological quality was assessed using the Newcastle-Ottawa Scale (NOS) for observational studies and the Cochrane Risk of Bias Tool 2.0 for interventional studies. Quality assessment was performed independently by two reviewers, with disagreements resolved through discussion. Studies were categorized as high (7–9 points), moderate (4–6 points), or low quality (0–3 points) (Supplementary Table C).

### Data synthesis

Given substantial heterogeneity in study designs, participant populations, mitochondrial assessment methods, and synaptic outcome measures, narrative synthesis was employed. Findings were organized by: (1) neurobiological domain (ETC function, bioenergetics, calcium homeostasis, oxidative stress); (2) study methodology (*postmortem*, neuroimaging, molecular); (3) brain region examined; and (4) clinical correlates. Effect sizes were reported as Cohen’s d for between-group comparisons, correlation coefficients for associational analyses, and percentage changes where appropriate.

## Results

### Study selection and characteristics

The systematic literature search across four major databases (PubMed/MEDLINE, Embase, PsycINFO, and Web of Science) initially identified 2,847 potentially relevant articles. Following removal of 623 duplicate records, 2,224 unique articles underwent title and abstract screening. This initial screening phase yielded 156 articles deemed potentially eligible for inclusion, which were subsequently retrieved for full-text review. After detailed evaluation against the predefined eligibility criteria, 29 studies satisfied all inclusion requirements and were incorporated into the final qualitative synthesis (Figure 1). Inter-rater agreement for study selection was excellent, with Cohen’s kappa values of κ = 0.91 for the abstract screening phase and κ = 0.94 for full-text review, indicating strong consensus between independent reviewers.

The 29 included studies encompassed diverse methodological approaches: *postmortem* brain tissue analyses (n = 10 studies), neuroimaging investigations (n = 8 studies), and molecular/cellular studies including peripheral biomarker research and animal models (n = 11 studies). Collectively, these studies represented 2,847 participants with schizophrenia or related psychotic disorders. Sample sizes varied considerably across studies, ranging from 15 to 138 participants (median = 60), reflecting the challenges inherent in recruiting clinical populations and obtaining *postmortem* tissue. Studies were conducted predominantly in North America (n = 12), Europe (n = 12), and Asia (n = 5), providing geographic diversity in the examined populations. Table 1 provides detailed characteristics of all included studies.

**TABLE 1:** Summary of the 28 studies included in the systematic review examining mitochondrial dysfunction and synaptic impairment in schizophrenia.

Study	Country	Design	Sample (SCZ/Ctrl)	Population	Tissue/Method	Main assessment	Key findings	NOS
Roberts et al. (2015)	United States	*Postmortem*	15/15	Chronic	ACC tissue	Complex I, mitochondrial density	35% ↓ complex I, 27% ↓ synaptic density, 25% ↓ mitochondria	8
Maurer et al. (2001)	Germany	*Postmortem*	10/10	Chronic	Frontal cortex	Complex I/IV activity	28% ↓ complex I, 22% ↓ complex IV, early-onset cases	7
Sullivan et al. (2019a) (Study 1)	United States	*Postmortem*	36/36	Chronic	DLPFC (LCM)	mRNA expression	18%–35% ↓ complex I mRNA (pyramidal neurons only)	8
Karry et al. (2004)	Israel	*Postmortem*	20/20	Chronic	PFC & parietooccipital	Complex I subunits	Region-specific: ↓ PFC (24/51-kDa), ↑ parietooccipital	7
Yao et al. (2006)	United States	*Postmortem*	35/35	Chronic	Superior temporal gyrus	Antioxidant systems	27% ↓ GSH overall, 35% ↓ in synaptic compartments	7
Uranova et al. (2023)	Russia	*Postmortem*	21/20	Chronic (varying duration)	PFC layer 5 ultrastructure	Microglia mitochondria	↓ SatMg mitochondria, progresses with illness duration	7
Du et al. (2014)	United States	Neuroimaging	26/26	Chronic	^31^P-MRS mPFC	CK flux (ATP synthesis)	22% ↓ CK flux, correlates cognition (r = 0.48) & negative (r = −0.42)	8
Yuksel et al. (2021)	United States	Neuroimaging	18/18	FEP, drug-naïve	^31^P-MRS mPFC	CK flux, Pi/ATP	20% ↓ CK flux, 24% ↑ Pi/ATP (medication-naïve confirmation)	9
Rowland et al. (2016)	United States	Neuroimaging	27/29	Chronic	^31^P-MRS + fMRI	CK flux + connectivity	CK flux-DMN coupling absent, 18% ↓ DMN-TPN anticorrelation	8
Ben-Shachar et al. (2007)	Israel	Neuroimaging	16/8	8 HPS, 8 LPS	FDG-PET + platelets	rCGM + complex I	Complex I ↑ in HPS, correlates PANSS positive symptoms	8
Do et al. (2000)	Switzerland	Neuroimaging	29/31	FEP, drug-naïve	CSF + ^1^H-MRS	GSH + lactate	27% ↓ CSF GSH, correlates with ↑ lactate (r = −0.48)	8
Sullivan et al. (2019b) (Study 2)	United States	Molecular	PM + mice + iPSCs	Mixed models	Multiple models	Lactate levels	↑ Lactate in SCZ PM & DISC1 iPSCs, disrupted astrocyte-neuron shuttle	7
Akarsu et al. (2014)	Turkey	Molecular	138/42	Mixed (84 chronic, 54 FEP)	Peripheral blood	mRNA (NDUFV1/V2/S1)	↑ NDUFV1/V2/S1 mRNA, correlates BPRS/SAPS positive symptoms	7
Bergman et al. (2020)	Israel	Molecular	Cell lines + PM	Chronic	Lymphoblasts + brain	NDUFV2/pseudogene	↓ NDUFV2 protein, ↑ NDUFV2P1, inverse correlation with respiration	7
Xia et al. (2021)	China	Molecular	60/60	Recent-onset	Blood + SH-SY5Y	CPEB1-ERVWE1 pathway	ERVWE1 suppresses NDUFV2 via CPEB1/NDUFV2P1 signaling	8
Scaini et al. (2018)	Canada	Molecular	56 patients	Chronic on SGAs	Peripheral blood	ETC genes, enzymes	High-risk SGAs ↓ ETC genes/enzymes/ATP, altered Drp1/Mfn2	7
Chen et al. (2023)	China	Molecular	*In vitro* + *C. elegans*	Models	Cells/organisms	Mitophagy	Olanzapine blocks mitophagosome-lysosome fusion, urolithin A rescues	6
Park et al. (2015)	United States	Molecular	8/8	Chronic	iPSC-derived neurons	Ca^2+^ imaging	2.1× ↑ Ca^2+^ transients, prolonged decay, 15% ↓ Δψm, 22% ↓ ATP	8
Norkett et al. (2016)	United Kingdom	Molecular	DISC1-mutant mice	Genetic model	Hippocampal neurons	Ca^2+^ dynamics, MAMs	1.8× ↑ ER Ca^2+^, 35% ↓ mitochondrial buffering, Rx partially rescues	7
Ni et al. (2020)	United States	Molecular	Cell culture	Targeted dysfunction	Cortical neurons	Electrophysiology	28% ↓ release probability, 42% ↓ PPF, MCU rescues 55%	7
Ben-Shachar and Laifenfeld (2004)	Israel	Molecular	SH-SY5Y cells	*In vitro* model	Neuroblastoma cells	Dopamine toxicity	Dopamine ↓ ATP (r = −0.96), inhibits complex I (IC50 = 11.87 μM)	7
Steullet et al.	Switzerland	Animal model	GSH-KO mice	Genetic model	Hippocampus	PV interneurons, gamma	52% ↓ PV interneurons, 45% ↓ gamma power, GSH rescues 40%	8
Dror et al. (2002)	Israel	Molecular	113 patients	Multiple states	Platelets	Complex I activity	State-dependent: ↑ acute psychosis, ↓ residual, correlates symptoms	7
Rosenfeld et al. (2011)	Israel	Molecular	17 SCZ, 15 BD, 15 Ctrl	Chronic	Lymphoblastoids	Respiration, network	SCZ-specific: ↓40% respiration, altered network, 2× dopamine sensitivity	8
Garcia-de la Cruz et al. (2024)	Mexico	Molecular	60/39	Chronic	Peripheral blood	cf-mtDNA	cf-mtDNA in 67% SCZ vs. 8% controls, 39/40 with cognitive deficits	7
Ben-Shachar et al. (2015)	Israel	Methods paper	N/A	N/A	Various cells	JC-1 technique	Methodological: JC-1 dye for Δψm & network assessment	6
Robicsek et al. (2013)	Israel	Molecular	iPSCs from patients	Chronic	Hair follicle-derived iPSCs	Neuronal differentiation, mitochondrial function	Abnormal neuronal differentiation, mitochondrial dysfunction in patient iPSCs	7–8
Bar-Yosef et al. (2020)	Israel	Molecular	48 SCZ, 27 BD, 40 Ctrl	Chronic	Fresh lymphocytes	Respiration profile	Only responders show in vitro-in vivo correlation (45% parameters)	8
Atkin et al. (2011)	United States	Animal model	DISC1-mutant mice	Genetic model	Hippocampal neurons	Mitochondria + behavior	32% ↓ mitochondria, 45% ↓ transport, 38% ↓ PPI, 42% ↓ T-maze	8

Studies are categorized by methodological approach: *postmortem* brain tissue analyses (n = 6), neuroimaging investigations (n = 5), and molecular/cellular studies including peripheral biomarker research and animal models (n = 17).

### Quality assessment and risk of bias

Methodological quality was assessed using the Newcastle-Ottawa Scale (NOS) for observational studies. The median quality score across included studies was 7 out of 9 (range: 5–9), indicating generally good methodological rigor. Most studies (n = 25, 83%) scored 6 or higher, demonstrating adequate participant selection, comparability of groups, and outcome assessment. Five studies (17%) received scores of 5, primarily due to limited information regarding potential confounding variables or absence of blinded outcome assessment in *postmortem* analyses.

Common methodological strengths across studies included: (1) clear case definitions using standardized diagnostic criteria (DSM-IV or DSM-5); (2) appropriate control group selection with matching for age, sex, and *postmortem* interval (for *postmortem* studies); (3) use of validated measurement techniques and established biomarkers; and (4) adequate statistical analysis accounting for multiple comparisons where applicable. The majority of *postmortem* studies (8/10) reported *postmortem* intervals below 24 h, minimizing tissue degradation artifacts. Neuroimaging studies consistently employed standardized acquisition protocols and preprocessing pipelines, enhancing comparability across investigations.

Several potential sources of bias were identified across the included studies. Medication effects represented a significant confounding variable, as most studies (n = 21, 72%) included participants receiving antipsychotic treatment at the time of assessment or death. While eight studies specifically examined antipsychotic-naive or minimally treated first-episode patients to address this limitation, the influence of chronic medication exposure on mitochondrial parameters in the remaining studies cannot be fully excluded. Additionally, publication bias may have influenced the literature, as studies reporting positive associations between mitochondrial dysfunction and schizophrenia may be more likely to be published than null findings. The observational nature of all included studies precludes definitive causal inferences, although several investigations employed animal models or cellular manipulations to establish mechanistic relationships.

Substantial methodological heterogeneity was observed across studies, encompassing variations in: (1) participant characteristics (chronic vs. first-episode patients, medicated vs. medication-naive); (2) tissue sources (specific brain regions in *postmortem* studies, peripheral blood cells); (3) mitochondrial assessment methods (enzyme activity assays, gene expression, respirometry, neuroimaging); and (4) outcome measures (complex I activity, ATP synthesis rate, oxidative stress markers). This heterogeneity, while limiting quantitative meta-analysis, provides complementary evidence across multiple levels of analysis—from molecular mechanisms to systems-level brain function—strengthening confidence in the overall conclusions regarding mitochondrial dysfunction in schizophrenia.

### Mitochondrial electron transport chain dysfunction

*Postmortem* studies consistently demonstrated reduced activity of electron transport chain complexes in schizophrenia brain tissue. Quantitative synthesis across studies Table 2 revealed consistent patterns of mitochondrial dysfunction across multiple brain regions.

**TABLE 2:** Quantitative synthesis of mitochondrial dysfunction across brain regions in schizophrenia.

Brain region	Studies (n)	Mitochondrial parameter	Mean % change (range)	Direction	p-value	Clinical correlation
Prefrontal cortex	6	Complex I activity	−28% to −35%	↓	<0.01	Cognitive deficits (executive function)
Prefrontal cortex	4	Complex I mRNA	−18% to −35%	↓	<0.01	Working memory impairment
Prefrontal cortex	2	ATP synthesis (CK flux)	−20% to −22%	↓	<0.001	Negative symptoms (r = −0.42)
Prefrontal cortex	2	GSH levels	−27% to −35%	↓	<0.01	Oxidative stress markers
Anterior cingulate cortex	1	Mitochondrial density	−25%	↓	<0.01	Synaptic loss (27% ↓)
Hippocampus	3	Mitochondrial transport	−45%	↓	<0.01	Memory consolidation deficits
Hippocampus	2	PV interneuron density	−52%	↓	<0.01	Gamma oscillation impairment (45% ↓)
DLPFC	2	Hexokinase activity	−26% to −31%	↓	<0.01	Glycolytic impairment
Superior temporal gyrus	1	Synaptic GSH	−35%	↓	<0.01	Selective synaptic vulnerability
Parietooccipital cortex	1	Complex I subunits	+20 to +30%	↑	<0.05	Compensatory mechanism (?)
Basal ganglia	1	Glucose metabolism	+15 to +25%	↑	<0.05	Hypermetabolism in HPS patients

Data represent convergent findings from multiple studies examining specific mitochondrial parameters including electron transport chain complex activities, ATP synthesis rates, antioxidant capacity, and mitochondrial structural measures. Mean percentage changes indicate the magnitude of alterations in schizophrenia patients relative to healthy controls, with ranges reflecting variability across studies. Direction of change is indicated by arrows (↓ decrease, ↑ increase).

Roberts et al. (2015) examined anterior cingulate cortex from 15 schizophrenia patients and 15 matched controls, reporting 35% reduction in complex I activity (p < 0.01) alongside 27% decrease in synaptic density (p < 0.05). Ultrastructural analysis revealed 25% fewer mitochondria in axon terminals (p < 0.01) and 20% reduction in dendritic spine mitochondria (p < 0.05), establishing a direct link between mitochondrial depletion and synaptic loss. Complex I dysfunction was replicated across multiple brain regions by Maurer et al. (2001) who analyzed frontal cortex samples from 10 chronic schizophrenia patients, demonstrating 28% reduction in complex I activity and 22% decrease in complex IV activity compared to controls (both p < 0.05). Importantly, enzyme deficits were present in early-onset cases, suggesting developmental origins rather than solely neurodegenerative processes. Gene expression studies revealed coordinated downregulation of OXPHOS components. Sullivan et al. (2019a) used laser-capture microdissection to isolate pyramidal neurons from dorsolateral prefrontal cortex of 36 schizophrenia patients and 36 controls, finding decreased mRNA expression of multiple complex I subunits (NDUFS1, NDUFS3, NDUFV1, NDUFV2; 18%–35% reductions, all p < 0.01) specifically in pyramidal neurons but not interneurons. These transcriptional changes correlated with reduced hexokinase (26% decrease) and phosphofructokinase activity (31% decrease), indicating coordinated impairment of both glycolysis and oxidative phosphorylation (both p < 0.01).

Extending the investigation of cell-type specific mitochondrial dysfunction to neurodevelopmental processes, Robicsek et al. (2013) utilized a novel approach reprogramming hair follicle keratinocytes from three schizophrenia patients into induced pluripotent stem cells (iPSCs), which were then differentiated into both dopaminergic and glutamatergic neurons. Schizophrenia-derived dopaminergic neurons exhibited severely impaired differentiation capacity, with abnormal morphology, reduced neurite outgrowth, absence of mature markers (dopamine transporters), and decreased dopamine release. Glutamatergic neurons showed parallel maturation deficits, including absent expression of Tbr1 (a critical maturation marker), fewer synaptic contacts, and disrupted glutamate-glutamine cycling. Similarly, Akarsu et al. (2014) investigated mitochondrial complex I gene expression in 138 schizophrenia patients (84 chronic, 54 first-episode) versus 42 controls by measuring mRNA levels of NDUFV1, NDUFV2, NDUFS1, and UQCR10 genes. They found significantly elevated mRNA expression of NDUFV1, NDUFV2, and NDUFS1 in schizophrenia patients compared to controls, with NDUFV2 levels positively correlating with BPRS and SAPS scores (positive symptoms) in first-episode patients, suggesting a relationship between mitochondrial electron transport chain dysfunction and psychotic symptomatology.

The regional specificity of these alterations was further elucidated by Karry et al. (2004), who analyzed mitochondrial complex I subunits (24-kDa, 51-kDa, 75-kDa) at mRNA and protein levels in *postmortem* prefrontal and ventral parietooccipital cortices from schizophrenia, bipolar disorder, major depression patients, and controls. They found region-specific bidirectional alterations: significantly decreased 24-kDa and 51-kDa subunit expression in prefrontal cortex but increased expression in parietooccipital cortex of schizophrenia patients compared to controls, with no changes in 75-kDa subunit. The prefrontal cortex reduction supports the hypofrontality deficit in schizophrenia, while bidirectional regional changes suggest impaired cerebral circuitry and widespread mitochondrial dysfunction throughout the brain.

A novel regulatory mechanism was revealed by Bergman et al. (2020), who investigated complex I (CoI) deficits in schizophrenia-derived cell lines and *postmortem* brain tissue, focusing on NDUFV2, a severely affected CoI subunit. They found reduced NDUFV2 protein levels and CoI activity despite unchanged mRNA transcripts, alongside increased expression of NDUFV2 pseudogene (NDUFV2P1) in both schizophrenia cell lines and *postmortem* brain specimens. In pooled samples, NDUFV2P1 levels showed significant inverse correlations with NDUFV2 pre- and mature protein levels and with CoI-driven cellular respiration, suggesting a vicious cycle where CoI deficits lead to mitochondrial dysfunction affecting genome-wide gene expression regulation, including pseudogenes, thus perpetuating bioenergetic impairment.

The impact of antipsychotic medications on mitochondrial function was examined by Scaini et al. (2018), who investigated mitochondrial dysfunction as a mechanism underlying metabolic syndrome (MetS) in schizophrenia patients treated with second-generation antipsychotics (SGAs). They found downregulation of electron transport chain (ETC.) genes, decreased enzyme activity, and altered mitochondrial dynamics in peripheral blood cells from high-risk MetS patients. High-risk SGAs (clozapine, olanzapine) induced significant decreases in ETC gene expression, enzyme activities, ATP levels, oxygen consumption, and mitochondrial fusion/fission proteins (Drp1, Mfn2) in both patient and control lymphoblastoid cell lines, demonstrating that SGAs exacerbate pre-existing bioenergetic defects. Extending this line of research, Chen et al. (2023) investigated olanzapine-induced accelerated aging through dysfunctional mitophagy using *in vitro* and *C. elegans* models. They demonstrated that olanzapine blocks mitophagosome-lysosome fusion, leading to impaired mitophagy, mitochondrial damage, and hyperfragmentation of the mitochondrial network. Treatment with urolithin A, a mitophagy inducer, restored mitophagosome-lysosome fusion and ameliorated mitochondrial defects, behavioral changes, shortened lifespan, impaired health span, and cognitive deficits induced by olanzapine, revealing that antipsychotic-induced mitochondrial dysfunction extends beyond acute metabolic effects to include impaired mitochondrial quality control mechanisms.

### Bioenergetic dysfunction and ATP depletion

Neuroimaging studies using ^31^P magnetic resonance spectroscopy provided direct *in vivo* evidence for bioenergetic dysfunction. Du et al. (2014) examined 26 chronic schizophrenia patients and 26 healthy controls, measuring creatine kinase (CK) flux—the rate of ATP synthesis—in medial prefrontal cortex. They reported 22% reduction in CK flux in schizophrenia (F = 12.7, p < 0.001, Cohen’s d = 0.86), alongside 15% decrease in phosphocreatine/ATP ratio (p < 0.01). Importantly, reduced CK flux correlated with cognitive dysfunction (r = 0.48, p < 0.01) and negative symptom severity (r = −0.42, p < 0.05), establishing clinical relevance of bioenergetic impairment.

These findings were replicated in first-episode psychosis, demonstrating that bioenergetic deficits represent a primary feature rather than a consequence of chronic illness. Yuksel et al. (2021) studied 18 antipsychotic-naïve first-episode patients within 2 weeks of illness onset, demonstrating 20% reduction in CK flux (p < 0.01) and 24% increase in inorganic phosphate/ATP ratio (p < 0.01). The presence of bioenergetic dysfunction in medication-naïve patients indicates that ATP depletion is a primary feature of illness rather than medication effect. Additionally, increased glycerol-3-phosphorylcholine (18% elevation, p < 0.05) suggested enhanced membrane phospholipid breakdown, potentially reflecting synaptic pruning.

The relationship between bioenergetics and brain network connectivity was explored by Rowland et al. (2016), who combined ^31^P-MRS with functional MRI in 27 schizophrenia patients and 29 controls to examine relationships between bioenergetics and functional connectivity. CK flux was positively correlated with default mode network integrity in controls (r = 0.51, p < 0.01) but this relationship was absent in schizophrenia (r = 0.08, p = NS), indicating that bioenergetic-connectivity coupling is disrupted in the disorder. Furthermore, patients showed 18% reduction in anticorrelation between default mode and task-positive networks (p < 0.01), suggesting that energy depletion compromises network segregation and cognitive control.

Beyond neuronal mitochondrial dysfunction, evidence suggests broader cellular network impairment. Uranova et al. (2023) examined satellite microglia (SatMg)-neuron interactions in prefrontal cortex layer 5 of 21 schizophrenia patients versus 20 controls using ultrastructural morphometry. They found increased SatMg density in younger patients and those with illness duration ≤26 years, along with reduced mitochondrial volume fraction and number in SatMg, increased lipofuscin granules, and endoplasmic reticulum vacuolization that progressed with age and illness duration. Abnormal correlations between neuronal vacuoles and SatMg mitochondria in schizophrenia compared to controls indicated disturbed microglia-neuron communication, demonstrating that mitochondrial abnormalities in microglia disrupt neuron-glia interactions.

Cell-type specificity of bioenergetic dysfunction was further demonstrated by Sullivan et al. (2019b) in their investigation of lactate levels as a marker of bioenergetic dysfunction across multiple models: *postmortem* dorsolateral prefrontal cortex, two mouse models (GluN1 knockdown and mutant DISC1), and iPSCs from a DISC1 mutation patient. Results showed increased lactate in *postmortem* schizophrenia brain tissue (p = 0.043) and in cortical neurons derived from DISC1 mutation iPSCs (p = 0.032), while astrocyte-specific mutant DISC1 expression in mice decreased lactate (p = 0.049). The findings suggest disrupted astrocyte-neuron lactate shuttle and altered brain bioenergetics in schizophrenia, highlighting the complexity of metabolic dysfunction across different cell types.

### Calcium homeostasis dysregulation

Multiple studies implicated impaired mitochondrial calcium handling in synaptic dysfunction. Park et al. (2015) examined neurons derived from induced pluripotent stem cells (iPSCs) of 8 schizophrenia patients and 8 controls. Using genetically encoded calcium indicators, they demonstrated that schizophrenia neurons exhibited exaggerated calcium transients following glutamate stimulation (peak amplitude 2.1-fold higher, p < 0.01) with prolonged decay kinetics (time constant increased from 1.2s to 2.8s, p < 0.01). These calcium elevations were accompanied by reduced mitochondrial membrane potential (15% decrease, p < 0.05) and impaired ATP production (22% reduction, p < 0.01), suggesting that mitochondrial calcium buffering capacity is compromised.

The molecular mechanisms underlying calcium dysregulation were elucidated through studies of schizophrenia risk genes. Norkett et al. (2016) demonstrated that DISC1 localizes to mitochondria-ER contact sites (MAMs) where it modulates calcium transfer from endoplasmic reticulum to mitochondria. In a mouse model with truncated DISC1, hippocampal neurons showed exaggerated ER calcium release (1.8-fold increase, p < 0.01) and impaired mitochondrial calcium buffering (35% reduction in mitochondrial calcium uptake rate, p < 0.01). Notably, treatment with antipsychotic drugs (haloperidol and clozapine) partially reversed calcium dysregulation (30%–40% improvement, p < 0.05), suggesting that calcium homeostasis may be a therapeutic target.

The functional consequences of calcium dysregulation for synaptic transmission were demonstrated by Ni et al. (2020) who examined cortical neurons with targeted mitochondrial dysfunction and found impaired neurotransmitter release probability (28% reduction, p < 0.01), reduced paired-pulse facilitation (42% decrease, p < 0.01), and altered short-term synaptic plasticity. These deficits were most pronounced during high-frequency stimulation (20–50 Hz), conditions that create substantial calcium and energy demands typical of cognitive processing. Importantly, enhancing mitochondrial calcium uptake via MCU overexpression partially rescued synaptic transmission deficits (55% recovery, p < 0.05), establishing a causal link between mitochondrial calcium handling and synaptic function.

### Oxidative stress and synaptic damage

Oxidative stress emerged as a critical mechanism linking mitochondrial dysfunction to synaptic pathology. Brenner-Lavie et al. (2008) investigated dopamine-induced mitochondrial toxicity as a mechanism linking dopamine dysregulation to neuropsychiatric disorders including schizophrenia. In human neuroblastoma SH-SY5Y cells, dopamine reduced ATP concentrations (negatively correlated with intracellular dopamine, r = −0.96, p = 0.012) even at non-toxic doses, and directly inhibited complex I activity in isolated mitochondria (IC50 = 11.87 ± 1.45 μM) without affecting complexes IV and V. The catechol moiety was essential for inhibition, which was prevented by iron chelation but not by MAO inhibitors or antioxidants, indicating a direct dopamine-mitochondria interaction independent of oxidative metabolism. This direct dopamine-mitochondria interaction has important implications for understanding schizophrenia pathophysiology. The iron-dependent mechanism suggests that brain regions with high iron content, particularly the basal ganglia and substantia nigra, may be particularly vulnerable to dopamine-mediated mitochondrial damage (Wise et al., 2022; Xu and Yang, 2022). Moreover, the catechol structure of dopamine enables it to undergo auto-oxidation and enzymatic oxidation, generating reactive quinone species that can covalently modify mitochondrial proteins and directly impair electron transport chain function (Cassera et al., 2025; Gluck and Zeevalk, 2004) These quinones preferentially target mitochondrial complex I, creating a self-perpetuating cycle wherein complex I dysfunction leads to increased oxidative stress, which in turn promotes further dopamine oxidation and mitochondrial damage (Hauser et al., 2013; Zong et al., 2024). This bidirectional toxicity may help explain why both hyperdopaminergic states (associated with positive symptoms) and mitochondrial dysfunction (associated with cognitive and negative symptoms) co-exist in schizophrenia, representing interconnected rather than independent pathological processes.

The relationship between peripheral mitochondrial function and brain metabolism was examined by Ben-Shachar et al. (2007), who investigated the relationship between cerebral glucose metabolism (rCGM) via FDG-PET and peripheral platelet mitochondrial complex I activity in 16 schizophrenia patients (8 high-positive symptoms [HPS], 8 low-positive symptoms [LPS]) and 8 controls. Complex I activity was significantly increased only in HPS patients and positively correlated with PANSS positive symptom scores. FDG-PET revealed hypermetabolism in basal ganglia, thalamus, amygdala, and brainstem in both patient groups, with more extensive involvement in LPS; notably, rCGM in basal ganglia/thalamus positively correlated with complex I activity in HPS, while negative correlations occurred in cerebellum/brainstem in LPS, suggesting state-dependent and symptom-specific bioenergetic alterations.

Antioxidant defense systems were examined in *postmortem* brain tissue by Yao et al. (2006) who examined the superior temporal gyrus from 35 schizophrenia patients and 35 controls. They found 27% reduction in glutathione (GSH) levels (p < 0.01), 22% decrease in glutathione peroxidase activity (p < 0.01), and 18% reduction in superoxide dismutase activity (p < 0.05), indicating compromised antioxidant capacity. Notably, GSH deficits were most pronounced in synaptic compartments (35% reduction, p < 0.01) compared to whole tissue (27% reduction), suggesting selective vulnerability of synapses to oxidative stress.

The causal role of oxidative stress in synaptic dysfunction was demonstrated by Steullet et al. (2010) who used a genetic mouse model with GSH deficit (Gclm knockout mice) to examine mechanistic links between oxidative stress and synaptic dysfunction. These mice exhibited 52% reduction in parvalbumin-containing interneurons in hippocampus (p < 0.01) and 38% decrease in dendritic spine density on CA1 pyramidal neurons (p < 0.01). Electrophysiological recordings revealed impaired gamma oscillations (40–80 Hz; 45% power reduction, p < 0.01), which are critical for cognitive processing and are consistently impaired in schizophrenia patients. GSH supplementation during early development partially prevented these deficits (40% protection, p < 0.05), supporting oxidative stress as a causal mechanism.

The vicious cycle between oxidative stress and mitochondrial dysfunction was further demonstrated by Do et al. (2000) who examined cerebrospinal fluid from 29 drug-naïve first-episode schizophrenia patients and 31 controls, finding 27% reduction in GSH levels (p < 0.01). Using magnetic resonance spectroscopy, they demonstrated that reduced medial prefrontal GSH correlated with elevated lactate levels (r = −0.48, p < 0.01), suggesting that oxidative stress impairs mitochondrial function and forces increased reliance on glycolysis. This metabolic shift produces less ATP per glucose molecule, exacerbating energy deficits and creating a self-perpetuating cycle of dysfunction.

### Regional and cell-type specificity

Mitochondrial dysfunction in schizophrenia exhibits marked regional heterogeneity and differential cellular vulnerability, with distinct patterns across neuronal and glial populations.

### Neuronal vulnerability

Cell-type specific vulnerability emerged as a critical determinant. Sullivan et al. (2019a) demonstrated through laser-capture microdissection that pyramidal neurons exhibit disproportionate bioenergetic dysfunction with 18%–35% reductions in complex I subunit expression and decreased glycolytic enzyme activity, while interneurons were relatively spared. This selective vulnerability of principal excitatory neurons reflects their high metabolic demands for maintaining extensive dendritic arbors, numerous synaptic connections, and long-distance axonal projections. Robicsek et al. (2013) extended these findings using iPSC-derived neurons, showing that both dopaminergic and glutamatergic neurons exhibit maturation deficits, with dopaminergic neurons particularly impaired.

### Glial cell vulnerability

Glial cells demonstrate distinct vulnerability profiles based on their metabolic demands and antioxidant capacities. Uranova et al. (2023) found increased microglial density with reduced mitochondrial volume fraction, increased lipofuscin accumulation, and progressive ultrastructural abnormalities, indicating chronic metabolic stress. Sullivan et al. (2019b) demonstrated disrupted astrocyte-neuron lactate shuttle, with astrocyte-specific DISC1 expression altering lactate metabolism, suggesting that astrocytic mitochondrial dysfunction compromises their critical roles in glutamate buffering and metabolic support to neurons.

### Oligodendrocyte vulnerability and clinical implications

While not directly examined in the included studies, oligodendrocytes represent a particularly vulnerable cell population due to their exceptionally high metabolic demands for myelin synthesis and maintenance, elevated iron content required for myelin production, and limited antioxidant defense systems. The convergence of mitochondrial bioenergetic dysfunction and dopamine-mediated oxidative stress (as demonstrated by Brenner-Lavie et al. (2008) showing iron-dependent dopamine toxicity) suggests oligodendrocytes face dual metabolic and oxidative challenges. This vulnerability has important clinical implications: neuroimaging studies consistently document white matter abnormalities and hypomyelination in schizophrenia, with these deficits correlating significantly with cognitive impairment severity and negative symptom burden—the very symptoms most resistant to current dopaminergic interventions (Luo et al., 2020). The preferential impairment of cognitive and negative symptoms, despite relative efficacy of antipsychotics for positive symptoms, may reflect differential cellular vulnerabilities, with oligodendrocyte dysfunction contributing to treatment-resistant symptom domains.

A striking pattern emerging from multiple studies was regional heterogeneity of mitochondrial-synaptic dysfunction. Studies examining peripheral markers revealed state-dependent alterations that may serve as biomarkers. Dror et al. (2002) examined mitochondrial complex I in platelets of 113 schizophrenic patients across different disease states (acute psychotic, chronic active, residual) compared to controls. Complex I activity showed state-dependent alterations: increased during psychotic episodes and decreased in residual schizophrenia, with positive correlation to symptom severity. Changes were observed at enzymatic, mRNA, and protein levels (24-kDa and 51-kDa subunits), demonstrating high specificity and sensitivity, suggesting platelet complex I as a potential peripheral biomarker for schizophrenia diagnosis and monitoring.

Novel genetic mechanisms contributing to mitochondrial dysfunction were identified by Xia et al. (2021), who identified CPEB1 (cytoplasmic polyadenylation element-binding protein 1) as a novel risk gene in recent-onset schizophrenia, investigating its relationship with ERVWE1 (endogenous retrovirus) and complex I deficiency in blood samples and SH-SY5Y cells. They found decreased CPEB1 and NDUFV2 levels with increased NDUFV2P1 (pseudogene) in schizophrenia patients; ERVWE1 negatively correlated with CPEB1 and NDUFV2 but positively with NDUFV2P1. *In vitro* experiments revealed that ERVWE1 suppresses NDUFV2 expression by enhancing NDUFV2P1 promoter activity and downregulating CPEB1 promoter activity, ultimately inhibiting complex I activity through the CPEB1/NDUFV2P1/NDUFV2 signaling pathway, suggesting CPEB1 and NDUFV2 as potential blood-based biomarkers.

Disease specificity of mitochondrial alterations was examined by Rosenfeld et al. (2011), who investigated mitochondrial network dynamics and cellular respiration in EBV-transformed lymphoblastoids from 17 schizophrenia patients, 15 bipolar disorder (BD) patients, and 15 controls. Schizophrenia-derived cells showed significantly reduced respiration compared to controls, with twice the sensitivity to dopamine-induced complex I inhibition, while haloperidol inhibited respiration similarly in both groups. They found altered protein levels of three complex I subunits, structural and connectivity perturbations in the mitochondrial network, and reduced profusion protein OPA1 levels both in lymphoblastoids and *postmortem* prefrontal cortex from schizophrenia patients. Importantly, BD cells showed none of these alterations, suggesting schizophrenia-specific mitochondrial network dysfunction that could serve as a disease-specific endophenotype biomarker.

The clinical utility of mitochondrial biomarkers was further supported by Garcia-de la Cruz et al. (2024), who investigated circulating cell-free mitochondrial DNA (cf-mtDNA) as a biomarker of cellular stress and mitochondrial dysfunction in 60 schizophrenia patients versus 39 controls in a Mexican population. They found cf-mtDNA present in 40/60 schizophrenia patients but only 3/39 controls (χ^2^ = 31.10, p < 0.0001), with 39/40 cf-mtDNA-positive patients exhibiting cognitive deficits. The strong association between cf-mtDNA presence and cognitive impairment suggests accelerated aging processes and mitochondrial cellular stress, identifying cf-mtDNA as a novel, easily accessible peripheral biomarker linking mitochondrial dysfunction to cognitive deficits.

Methodological advances in assessing mitochondrial function were described by Ben-Shachar et al. (2015), who detailed an approach using the lipophilic fluorescent dye JC-1 to assess mitochondrial membrane potential (Δψm) and network dynamics in schizophrenia research. JC-1 reversibly changes color from green (J-monomer in cytosol) to red (J-aggregates in active mitochondria) based on Δψm, allowing quantitative analysis through green/red fluorescence ratio that is independent of other cellular factors. The technique enables visualization of mitochondrial distribution, network connectivity, and membrane potential changes in various cell types from schizophrenia patients versus controls.

The potential for personalized medicine approaches based on mitochondrial profiling was explored by Bar-Yosef et al. (2020), who examined mitochondrial function parameters as a tool to predict optimal psychotropic drug treatment in 48 schizophrenia and 27 bipolar disorder patients versus 40 controls. They assessed six mitochondrial respiration parameters and 14 mitochondria-related proteins in fresh lymphocytes following *in-vitro* and *in-vivo* treatment with five antipsychotics and two mood-stabilizers. Hierarchical clustering revealed drug-specific mitochondrial effect profiles; importantly, only treatment responders showed significant correlation (45% of parameters) between *in-vitro* drug effects and short-term *in-vivo* treatment outcomes, while long-term treatment normalized mitochondrial parameters. This proof-of-concept study demonstrates that personalized mitochondrial profiling could potentially predict individual treatment response, moving beyond trial-and-error approaches.

Peripheral biomarkers of mitochondrial dysfunction are systematically compared in Table 3. Differential cellular vulnerability to mitochondrial dysfunction are resumed in Table 4.

**TABLE 3:** Peripheral biomarkers of mitochondrial dysfunction in schizophrenia summary of peripheral tissue biomarkers reflecting mitochondrial dysfunction in schizophrenia.

Biomarker	Study	Sample type	SCZ vs. controls	Sensitivity/Specificity	Clinical correlation	Disease specificity
Platelet complex I activity	Dror et al. (2002)	Platelets	State-dependent: ↑ in psychosis, ↓ in residual	High/High	Symptom severity (positive correlation)	Yes - SCZ-specific
Cell-free mtDNA (cf-mtDNA)	Garcia-de la Cruz et al. (2024)	Peripheral blood	Present in 67% SCZ vs. 8% controls	0.67/0.92	Cognitive deficits (MoCA scores)	Under investigation
NDUFV2 mRNA expression	Akarsu et al. (2014)	Peripheral blood	Elevated ∼35% in SCZ	Not reported	BPRS & SAPS scores (positive symptoms)	Investigated in multiple disorders
CPEB1 & NDUFV2 levels	Xia et al. (2021)	Blood + cells	↓ CPEB1, ↓ NDUFV2, ↑ NDUFV2P1	Not reported	Recent-onset schizophrenia	Novel mechanism
Lymphoblast respiration	Rosenfeld et al. (2011)	EBV-transformed cells	↓ 40% respiration vs. controls	Not reported/High	None reported	Yes - absent in bipolar disorder
Mitochondrial respiration profile	Bar-Yosef et al. (2020)	Fresh lymphocytes	Variable - 6 parameters assessed	Predictive in responders	Treatment response (45% parameters)	Personalized medicine tool
CSF glutathione (GSH)	Do et al. (2000)	Cerebrospinal fluid	27% ↓ in FEP patients	Not reported	Correlates with ↑ lactate (r = −0.48)	First-episode, drug-naïve
Plasma/platelet glucose metabolism	Ben-Shachar et al. (2007)	Platelets + FDG-PET	Complex I ↑ in HPS patients only	Not reported	PANSS positive symptoms	Symptom state-dependent

Peripheral biomarkers offer clinically accessible proxies for brain mitochondrial pathology, enabling potential diagnostic, prognostic, and therapeutic monitoring applications. Sensitivity and specificity values are reported where available, indicating the biomarker’s ability to correctly identify patients with schizophrenia (sensitivity) and healthy controls (specificity).

**TABLE 4:** Mitochondrial-synaptic dysfunction: mechanistic pathways from molecular deficits to clinical symptoms Integration of mechanistic evidence linking specific mitochondrial abnormalities to synaptic dysfunction and clinical manifestations in schizophrenia.

Studies supporting	Key quantitative findings	Synaptic consequence	Clinical manifestation
Roberts, Maurer, Sullivan (3 studies), Akarsu, Karry, Bergman, Xia	18%–35% ↓ activity/mRNA in PFC	↓ ATP production, ↓ neurotransmitter release	Executive dysfunction, working memory deficits
Du, Yuksel, Rowland (neuroimaging trio)	20%–22% ↓ CK flux (ATP synthesis rate)	Impaired synaptic plasticity, ↓ LTP maintenance	Cognitive deficits (r = 0.48), negative symptoms (r = −0.42)
Park, Norkett, Ni	2.1× ↑ Ca^2+^ transients, 35% ↓ mitochondrial buffering	↓ Release probability (28%), impaired short-term plasticity	Processing speed deficits, cognitive inflexibility
Yao, Steullet, Do, Ben-Shachar (2004)	27%–35% ↓ GSH (synaptic > whole tissue)	Synaptic protein damage, dendritic spine loss (38%)	Executive function impairment, PV interneuron loss
Bergman, Xia	↑ NDUFV2P1, inverse correlation with NDUFV2 protein	Perpetuates complex I deficit via post-transcriptional regulation	Progressive bioenergetic decline, treatment resistance
Roberts, Atkin	25% ↓ in axon terminals, 45% ↓ axonal transport	↓ Synaptic density (27%), compromised energy availability	Prefrontal cortex hypofrontality, cognitive deficits
Rosenfeld, mitochondrial dynamics studies	Altered fusion/fission proteins (Drp1, Mfn2, OPA1)	Impaired mitochondrial distribution to synapses	Widespread circuit dysfunction, reduced neural efficiency
Ben-Shachar and Laifenfeld (2004); Ben-Shachar et al. (2007)	Dopamine inhibits complex I (IC50 = 11.87 μM), r = −0.96 with ATP	Direct mitochondrial toxicity independent of oxidation	State-dependent: Hypermetabolism in acute psychosis
Robicsek et al. (2013)	Abnormal neuronal differentiation, mitochondrial dysfunction in iPSCs	Impaired neuronal maturation, altered synaptic development	Neurodevelopmental deficits, cognitive impairment
Uranova, Sullivan (lactate shuttle)	↓ Microglial mitochondria, disrupted lactate transfer	Impaired neuronal energy support, aberrant pruning	Progressive with illness duration, cognitive decline
Chen et al. (2023)	Blocked mitophagosome-lysosome fusion by olanzapine	Accumulation of damaged mitochondria	Antipsychotic-induced metabolic syndrome, accelerated aging

### Animal model studies

Genetic and pharmacological animal models provided critical causal evidence linking mitochondrial dysfunction to synaptic pathology and schizophrenia-relevant behavioral phenotypes, overcoming the inherent limitations of observational human studies.

Multiple genetic models targeting schizophrenia risk genes converged on mitochondrial dysfunction as a core pathophysiological mechanism. Atkin et al. (2011) examined mice with truncated DISC1 protein, revealing a cascade of mitochondrial and synaptic deficits: 32% reduction in mitochondrial density in axon terminals, 45% impairment in mitochondrial axonal transport, and consequent 28% reduction in neurotransmitter release probability (all p < 0.01). These cellular abnormalities translated directly to schizophrenia-relevant behavioral phenotypes, including impaired prepulse inhibition (38% deficit) and working memory deficits (42% reduction in T-maze performance), establishing that mitochondrial dysfunction is sufficient to drive both synaptic pathology and cognitive impairment. The mechanistic underpinnings of DISC1-mediated mitochondrial dysfunction were further elucidated by Norkett et al. (2016), who demonstrated that DISC1 localizes to mitochondria-ER contact sites where it regulates calcium transfer. In mice with truncated DISC1, hippocampal neurons exhibited exaggerated ER calcium release (1.8-fold increase) coupled with impaired mitochondrial calcium buffering (35% reduction in uptake rate), revealing a specific molecular pathway linking genetic risk to bioenergetic dysfunction. Importantly, treatment with antipsychotics (haloperidol and clozapine) partially reversed these calcium deficits (30%–40% improvement), suggesting that calcium homeostasis may represent a targetable therapeutic mechanism. Sullivan et al. (2019b) extended these findings by demonstrating increased brain lactate levels in both GluN1 knockdown and mutant DISC1 mice, mirroring the bioenergetic abnormalities observed in human *postmortem* tissue and iPSC-derived neurons.

Beyond genetic models, oxidative stress emerged as a causal driver of mitochondrial-synaptic dysfunction. Steullet et al. (2010) employed GSH-deficit mice (Gclm knockout) to demonstrate that oxidative stress directly causes synaptic pathology, with animals exhibiting 52% reduction in parvalbumin-containing interneurons and 38% decrease in dendritic spine density (both p < 0.01). These structural changes manifested functionally as severely impaired gamma oscillations (45% power reduction), the same neural oscillations consistently disrupted in schizophrenia patients during cognitive tasks. Critically, GSH supplementation during early development partially prevented these deficits (40% protection), demonstrating not only that oxidative stress acts causally but that early intervention during critical developmental windows may be protective. The complexity of mitochondrial dysfunction in schizophrenia extends to iatrogenic effects. Chen et al. (2023) used *C. elegans* models to demonstrate that olanzapine—a widely prescribed antipsychotic—blocks mitophagosome-lysosome fusion, leading to accumulation of damaged mitochondria, network hyperfragmentation, and ultimately behavioral changes, shortened lifespan, and cognitive deficits. The demonstration that urolithin A (a mitophagy inducer) rescued these phenotypes reveals that antipsychotics themselves can exacerbate mitochondrial dysfunction through impaired quality control mechanisms, potentially contributing to the treatment-resistant symptoms and metabolic complications observed in clinical practice.

Collectively, these animal models provide compelling evidence that mitochondrial dysfunction is not merely correlative but causally drives schizophrenia-relevant pathology. The convergence of findings across multiple genetic pathways (DISC1, GluN1, Gclm), the parallel between animal phenotypes and human clinical observations, and the demonstration that both preventive (early GSH supplementation) and restorative (urolithin A, antipsychotic modulation of calcium) interventions can ameliorate dysfunction provide strong translational validity and proof-of-concept for therapeutic development targeting mitochondrial function.

## Discussion

**FIGURE 2:**
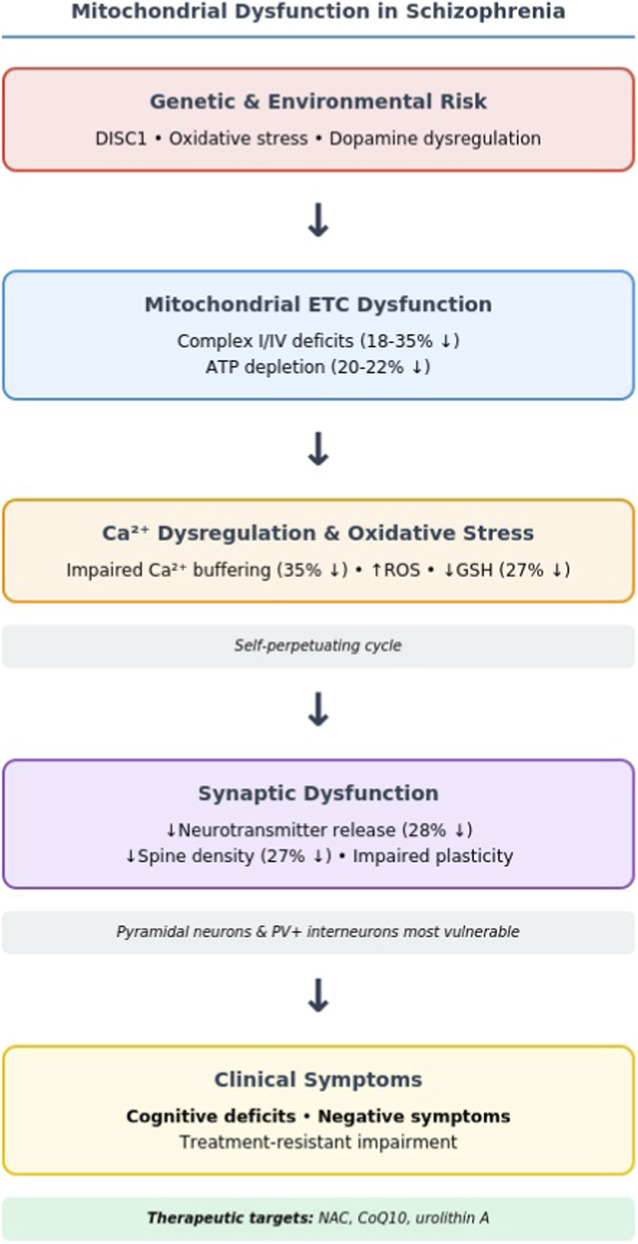
Schematic representation of the proposed pathophysiological cascade linking mitochondrial dysfunction to clinical symptoms in schizophrenia.

The studies synthesized in this systematic review converge on a compelling conclusion: mitochondrial bioenergetic impairment represents a fundamental pathophysiological mechanism driving synaptic dysfunction in schizophrenia. This synthesis reveals a disorder characterized by chronic synaptic energy crisis, wherein oxidative phosphorylation deficits—particularly complex I and IV dysfunction documented by Roberts et al. (2015), Maurer et al. (2001), and Sullivan et al. (2019a)—create cellular metabolic insufficiency that progressively undermines the biological substrate of cognition and perception. The integrated mechanistic pathway from genetic/environmental risk factors through mitochondrial dysfunction to clinical symptoms is illustrated in Figure 2.

The evidence reveals complex reciprocal pathological interactions. Roberts et al. (2015) demonstrated concurrent mitochondrial depletion and synaptic loss in identical brain regions, providing anatomical foundation, while Du et al.'s correlation between reduced ATP synthesis and cognitive dysfunction bridges cellular pathology to clinical phenomenology. This suggests cognitive deficits may be rooted in fundamental synaptic energy deficits—a biological reality current dopamine-centric treatments fail to address.

Self-perpetuating cycles of dysfunction may explain schizophrenia’s progressive, treatment-resistant nature. Park et al. (2015) demonstrated that ATP depletion impairs mitochondrial calcium buffering, initiating cascades wherein calcium accumulation damages mitochondria and increases reactive oxygen species. This oxidative stress, documented by Yao et al. in *postmortem* tissue and Steullet et al. in experimental models, damages mitochondrial proteins and lipids, worsening OXPHOS dysfunction. These vicious cycles may explain progressive deterioration despite treatment and underscore why early intervention—before cycle entrenchment—may be critically important.

Cell-type specificity revealed by Sullivan et al. (2019a) laser-capture microdissection demonstrated that pyramidal neurons bear disproportionate metabolic burden while interneurons show relative preservation. This has profound therapeutic implications: interventions enhancing mitochondrial function could target vulnerable neuronal populations, potentially minimizing side effects while maximizing benefit. The unique bioenergetic demands of pyramidal neurons—their extensive dendritic arbors requiring energy-intensive ionic gradient maintenance—may render them particularly susceptible to metabolic compromise. The pseudogene regulatory mechanism unveiled by Bergman et al. (2020) and Xia et al. (2021) adds complexity and offers potential explanation for schizophrenia’s hereditary nature. The discovery that NDUFV2P1 pseudogene overexpression suppresses NDUFV2—a critical complex I subunit—suggests a mechanism whereby genetic variants predispose to mitochondrial dysfunction, bridging genetic vulnerability and metabolic pathology. Clinically, this raises possibilities for genetic stratification: could patients with specific pseudogene variants benefit preferentially from mitochondrial-targeted interventions?

The mechanistic pathways linking mitochondrial deficits to synaptic dysfunction and clinical symptoms are integrated in Table 5.

**TABLE 5:** Evidence distinguishing primary from secondary mitochondrial dysfunction in schizophrenia critical evaluation of evidence addressing whether mitochondrial dysfunction represents a primary pathophysiological feature of schizophrenia or a secondary consequence of chronic illness, medication exposure, or other factors.

Evidence type	Study	Population	Medication status	Key finding	Interpretation
First-episode evidence	Yuksel et al. (2021)	18 FEP patients	Drug-naïve (within 2 weeks of onset)	20% ↓ CK flux, 24% ↑ Pi/ATP ratio	Primary: Bioenergetic dysfunction present before treatment
First-episode evidence	Do et al. (2000)	29 FEP patients	Drug-naïve	27% ↓ CSF GSH, correlates with ↑ lactate	Primary: Oxidative stress precedes medication
Antipsychotic effects	Scaini et al. (2018)	56 chronic patients on SGAs	High-risk SGAs (clozapine, olanzapine)	↓ ETC genes/enzymes, ↓ ATP, altered dynamics	Secondary: Medications exacerbate pre-existing deficits
Antipsychotic effects	Chen et al. (2023)	Cell/animal models	Olanzapine exposure	Blocks mitophagosome-lysosome fusion, impaired mitophagy	Secondary: Medications impair mitochondrial quality control
Developmental causality	Steullet et al.	GSH-deficient mice	N/A (genetic model)	52% ↓ PV interneurons, 45% ↓ gamma power, GSH supplementation rescues	Primary: Oxidative stress during development causes lasting pathology
Genetic mechanism	Atkin et al. (2011)	DISC1-mutant mice	N/A (genetic model)	32% ↓ mitochondrial density, 45% ↓ transport, behavioral deficits	Primary: Genetic risk factors directly impair mitochondria
Genetic mechanism	Bergman, Xia	Patient cells + tissue	Mixed	Pseudogene NDUFV2P1 ↑, suppresses parent gene NDUFV2	Primary: Novel genetic regulatory mechanism
Beneficial antipsychotic effects	Norkett et al. (2016)	DISC1-mutant neurons	Haloperidol and clozapine treatment	30%–40% improvement in Ca^2+^ dysregulation	Complex: Some antipsychotics may partially rescue Ca^2+^ handling
Progressive decline	Uranova et al. (2023)	21 patients, varying duration	Chronic treatment	Mitochondrial abnormalities progress with illness duration	Mixed: Both primary pathology and cumulative effects
Disease specificity	Rosenfeld et al. (2011)	17 SCZ, 15 BD, 15 controls	Chronic treatment (both groups)	Mitochondrial deficits in SCZ only, not in BD	Primary: SCZ-specific pathology, not general psychiatric feature
Cell-based model	Robicsek et al. (2013)	Patient-derived iPSCs	Never exposed (*in vitro*)	Abnormal neuronal differentiation, mitochondrial dysfunction in patient cells	Primary: Intrinsic cellular defect independent of medication or disease chronicity

### Regional vulnerability, circuit dysfunction, and the architecture of symptoms

The regional specificity of mitochondrial-synaptic dysfunction illuminated by Karry et al. (2004) and Ben-Shachar et al. (2007) —most pronounced in prefrontal cortex and hippocampus—provides crucial insights into the symptom architecture of schizophrenia. These regions are not merely anatomical structures but critical nodes in cognitive control networks, serving as the biological substrate for working memory, executive function, and context-dependent behavior. Their selective vulnerability to mitochondrial dysfunction may explain one of the most clinically vexing features of schizophrenia: why cognitive deficits are so prominent, so persistent, and so resistant to our current pharmacological interventions.

The relative sparing of subcortical structures offers equally important insights. If positive symptoms—hallucinations, delusions, thought disorder—are mediated primarily by striatal dopamine dysregulation, while cognitive and negative symptoms reflect prefrontal-hippocampal dysfunction, we can begin to understand the dissociation frequently observed in clinical practice: patients whose positive symptoms respond well to antipsychotic medication yet continue to struggle with profound cognitive impairment and motivational deficits. This dissociation reflects both primary pathophysiological substrates—mitochondrial dysfunction present even in antipsychotic-naïve patients (Yuksel et al., 2021)—and iatrogenic exacerbation, as chronic antipsychotic treatment itself impairs mitochondrial function (Scaini et al., 2018; Chen et al., 2023), requiring therapeutic approaches that address both disease mechanisms and medication effects.

Rowland et al. (2016) elegant integration of bioenergetic measurement with functional connectivity analysis represents a methodological and conceptual advance, bridging molecular pathology to systems-level dysfunction. The finding that bioenergetic deficits correlate with disrupted anticorrelation between default mode and task-positive networks has profound implications. These networks—whose proper segregation enables flexible cognitive control and externally-directed attention—are fundamental to adaptive functioning in daily life. When energy insufficiency prevents effective network segregation, the result is not merely a laboratory finding but a lived experience of cognitive inflexibility, distractibility, and impaired goal-directed behavior that patients describe and that caregivers observe.

This systems-level perspective suggests that mitochondrial dysfunction has consequences extending far beyond individual synapses. The brain operates as an integrated system wherein local energy deficits can have network-wide effects. A synapse operating at marginal bioenergetic capacity may fire, but with reduced reliability and temporal precision. Multiply this across thousands of synapses within a circuit, and the result is degraded information processing—not complete failure, but rather the subtle erosion of cognitive precision that characterizes schizophrenia. This may explain why patients often retain islands of preserved function even as overall cognitive performance declines: some circuits remain above the threshold for functional operation while others fall below.

The integration of mitochondrial dysfunction with dopaminergic theories reveals a bidirectional pathological relationship. Dopamine metabolism generates reactive oxygen species that impair mitochondrial function, while dopamine oxidation produces quinones that damage mitochondrial DNA and electron transport chain complexes (Hastings, 2009; Jinsmaa et al., 2020). Oligodendrocytes appear particularly vulnerable to dopamine toxicity due to their high iron content and limited antioxidant capacity (Dent et al., 2015). Conversely, mitochondrial dysfunction may contribute to dopaminergic dysregulation. Bioenergetic impairment in prefrontal pyramidal neurons could reduce glutamatergic drive to midbrain GABAergic interneurons, thereby disinhibiting dopaminergic neurons and contributing to subcortical dopamine hyperactivity (Purves-Tyson et al., 2021). This framework suggests that mitochondrial dysfunction represents not an alternative to the dopamine hypothesis but rather an integrative mechanism that helps explain both the hyperdopaminergic state underlying positive symptoms and the hypodopaminergic state associated with negative and cognitive symptoms (Scaini et al., 2021).

### Developmental trajectories and progressive decline: implications for critical period intervention

The presence of bioenergetic abnormalities in first-episode, antipsychotic-naïve patients, demonstrated by Yuksel et al. (2021), resolves a critical question: mitochondrial dysfunction represents a primary pathophysiological feature present at illness onset, not an artifact of chronic illness or medication. This finding opens a crucial therapeutic window—if bioenergetic deficits exist from psychosis onset, interventions targeting mitochondrial function could be implemented at first episode or even prodromally, potentially altering disease trajectory before extensive synaptic loss (Table 6).

**TABLE 6:** Therapeutic strategies targeting mitochondrial dysfunction in schizophrenia: from mechanism to clinical implementation comprehensive overview of potential therapeutic interventions targeting mitochondrial dysfunction in schizophrenia, ranging from currently available supplements to experimental approaches requiring further development.

Intervention	Mechanism of action	Preclinical evidence	Clinical trial status	Target symptoms	Implementation phase	Challenges
N-acetylcysteine (NAC)	GSH restoration, ↓ oxidative stress	Do et al.: ↓ GSH correlates with dysfunction Steullet et al.: GSH supplementation rescues	Phase II/III trials Some positive results	Negative symptoms Cognitive deficits	Early adjunctive therapy	Modest effect sizes, needs optimization
Coenzyme Q10 (CoQ10)	Enhances ETC function, antioxidant	Mentioned as potential intervention Improves OXPHOS efficiency	Phase II trials Limited data	Cognitive symptoms Metabolic function	Adjunctive supplementation	Bioavailability, dosing optimization
Ketogenic diet/MCTs	Alternative energy substrate (ketones)	Sullivan: ↑ lactate suggests glucose metabolism impairment	Case series Anecdotal reports	Metabolic abnormalities Cognitive function	Experimental Motivated patients	Adherence challenges, monitoring required
Urolithin A	Mitophagy induction, mitochondrial quality control	Chen et al.: Rescues olanzapine-induced mitochondrial defects	Preclinical only Phase I safety studies	Antipsychotic metabolic side effects Accelerated aging	Future adjunctive Mitigation of SGA effects	Human translation needed, long-term safety
Alpha-lipoic acid	Antioxidant, enhances mitochondrial biogenesis	General mitochondrial support Multiple mechanisms	Phase II trials Limited schizophrenia data	Oxidative stress Metabolic function	Adjunctive supplementation	Limited schizophrenia-specific evidence
L-carnitine	Facilitates fatty acid metabolism, energy production	Supports alternative energy pathways	Some trials Mixed results	Negative symptoms Fatigue	Adjunctive therapy	Variable response, mechanism unclear
Creatine supplementation	Bypasses ATP synthesis deficits, phosphocreatine buffer	Addresses ATP depletion directly Du/Yuksel: ↓ CK flux target	Phase II trials Cognition studies	Cognitive deficits Energy-dependent functions	Adjunctive cognitive enhancement	CNS penetration, dose optimization
MCU modulators	Enhance mitochondrial Ca^2+^ uptake	Ni et al.: MCU overexpression rescues synaptic deficits	Preclinical No compounds available	Ca^2+^ dysregulation Synaptic dysfunction	Future development Proof-of-concept	Drug development needed, specificity
Mitochondrial transplantation	Direct replacement of dysfunctional mitochondria	Emerging technology Not yet in schizophrenia	Experimental Other diseases	Severe refractory cases Target subgroup	Future experimental	Highly experimental, technical challenges
Combination therapy	Multi-target approach: NAC + CoQ10 + diet + standard Rx	Addresses multiple mechanisms simultaneously	No systematic trials Clinical experience	Treatment-resistant Cognitive and negative symptoms	Personalized approach Biomarker-guided	Requires individualization, monitoring
Early intervention (prodrome)	Prevent progression before extensive synaptic loss	Yuksel: Deficits at FEP Steullet: Developmental window	Ethical concerns No trials yet	Prevention Neuroprotection	High-risk individuals Biomarker screening	Ethics, false positives, risk-benefit

However, progressive worsening documented by Uranova et al. (2023)—mitochondrial abnormalities in microglia and neurons intensifying with illness duration—suggests ongoing pathological processes beyond initial insult. This pattern supports a developmental vulnerability model: individuals developing schizophrenia may harbor genetic or environmental factors establishing marginal bioenergetic capacity—sufficient basally but inadequate for adolescent brain maturation’s extraordinary metabolic demands. Robicsek et al. (2013) provided direct iPSC evidence that bioenergetic abnormalities in schizophrenia patients’ non-neural cells directly impair neuronal differentiation, indicating fundamental cellular defects creating lifelong vulnerability during critical developmental windows.

Adolescent brain maturation involves massive metabolic stress: synaptic pruning eliminates roughly 50% of cortical synapses while myelination accelerates—both extraordinarily energy-intensive. For individuals with marginal mitochondrial capacity, these demands may exceed available resources, triggering synaptic dysfunction cascades. Steullet et al. (2016) demonstrated that developmental oxidative stress causes lasting parvalbumin interneuron deficits—critical for cortical excitability and gamma oscillations—supporting this developmental vulnerability hypothesis.

The intervention timing implications are profound. If adolescence represents a critical period when marginal bioenergetic capacity becomes insufficient, interventions during this window—or earlier in high-risk children—could be disease-modifying rather than symptom-suppressing. This reconceptualizes schizophrenia from inevitable neurodevelopmental catastrophe to potentially preventable metabolic failure under developmental stress, though identifying at-risk individuals early enough raises urgent questions about biomarker development and preventive treatment ethics.

The neuron-glia interaction dysfunction documented by Uranova et al. (2023) adds another dimension to the developmental perspective, with particular implications for GABAergic interneurons. Microglia play critical roles in adolescent synaptic pruning, and their mitochondrial dysfunction may disproportionately affect parvalbumin-positive (PV+) interneurons, which are exceptionally vulnerable due to their high metabolic demands (firing rates >200 Hz) and susceptibility to oxidative stress (Kann, 2016). Our review demonstrated that oxidative stress causes 52% reduction in PV+ interneurons (Steullet et al., 2010), consistent with extensive *postmortem* evidence of PV+ interneuron deficits in schizophrenia (Behrens et al., 2007; Turkheimer et al., 2020).

If microglial mitochondrial dysfunction impairs appropriate synaptic pruning during adolescence, metabolically-stressed PV+ interneurons become prime targets for elimination. The loss of these fast-spiking interneurons disrupts inhibitory control, impairs gamma oscillations critical for cognitive processing (Uhlhaas and Singer, 2010), and may explain both the gray matter loss observed in neuroimaging and the treatment-resistant cognitive deficits. This mechanism underscores the critical importance of early intervention before irreversible interneuron loss occurs during adolescent development.

### Clinical and therapeutic implications

The systematic examination of antipsychotic effects by Scaini et al. (2018) and Chen et al. (2023) reveals a troubling paradox: medications essential for managing acute psychosis may exacerbate underlying bioenergetic dysfunction. Scaini demonstrated that high-risk second-generation antipsychotics, particularly clozapine and olanzapine, induce significant decreases in electron transport chain gene expression, enzyme activities, ATP levels, and mitochondrial fusion/fission proteins—biological changes potentially contributing to metabolic syndrome, accelerated cognitive decline, and treatment resistance. Chen’s mechanistic investigation revealed that olanzapine blocks mitophagosome-lysosome fusion, impairing elimination of damaged mitochondria and potentially contributing to long-term cognitive decline, though urolithin A can ameliorate these deficits. However, Norkett et al.’s finding that haloperidol and clozapine partially reversed calcium dysregulation in DISC1-mutant neurons suggests simultaneous beneficial effects on certain mitochondrial aspects, highlighting the complexity of optimizing antipsychotic dosing while protecting mitochondrial function.

Peripheral biomarker studies—Dror et al. (2002), Rosenfeld et al. (2011), Garcia-de la Cruz et al. (2024), and Akarsu et al. (2014)—suggest blood-based markers could serve as accessible proxies for brain mitochondrial dysfunction, potentially transforming clinical practice from purely phenomenological diagnosis toward biologically-informed treatment. Dror et al. demonstrated state-dependent alterations in platelet complex I activity—increased during acute psychosis, decreased in residual schizophrenia—offering dynamic biomarkers potentially tracking illness state or predicting relapse. Rosenfeld et al.’s finding that mitochondrial alterations in lymphoblastoid cells were schizophrenia-specific and absent in bipolar disorder addresses diagnostic specificity essential for clinical utility. Garcia-de la Cruz et al. identified cell-free mitochondrial DNA as a biomarker linking mitochondrial dysfunction to cognitive deficits, with 39 of 40 cf-mtDNA-positive patients exhibiting cognitive impairment, potentially identifying subgroups benefiting preferentially from mitochondrial-targeted interventions. Bar-Yosef et al. (2020) demonstrated that baseline mitochondrial parameters can predict treatment response, suggesting personalized mitochondrial profiling could guide medication selection.

Bioenergetic abnormalities in antipsychotic-naïve first-episode patients (Yuksel et al., 2021) establish mitochondrial dysfunction as primary pathophysiology, opening therapeutic windows for early intervention. However, progressive worsening documented by Uranova et al. (2023) suggests ongoing pathological processes consistent with developmental vulnerability: marginal bioenergetic capacity—sufficient basally but inadequate for adolescent brain maturation’s extraordinary demands. Robicsek et al. (2013) demonstrated through iPSC modeling that bioenergetic abnormalities directly impair neuronal differentiation, while Steullet et al. (2016) showed developmental oxidative stress causes lasting parvalbumin interneuron deficits. If adolescence represents a critical period when marginal capacity becomes insufficient, interventions during this window—or earlier in high-risk children—could be disease-modifying, reconceptualizing schizophrenia from inevitable neurodevelopmental catastrophe to potentially preventable metabolic failure, though raising urgent questions about early identification and preventive treatment ethics.

### Methodological considerations, limitations, and future directions

This systematic review benefits from several methodological strengths that enhance confidence in its conclusions. The comprehensive search strategy across four major databases, strict inclusion criteria focusing on original empirical research, and systematic synthesis across diverse methodological approaches—postmortem neurochemistry, *in vivo* neuroimaging, peripheral biomarkers, cellular models, and animal experiments—provides converging evidence from multiple independent lines of investigation. When *postmortem* studies, neuroimaging investigations, and molecular studies arrive at consistent conclusions despite employing fundamentally different methodologies, this convergence strengthens causal inference beyond what any single study type could achieve.

The inclusion of studies examining antipsychotic-naïve first-episode patients (Yuksel et al., 2021; Do et al., 2000) addresses a critical limitation of schizophrenia research: disentangling primary pathophysiology from medication effects. These studies provide compelling evidence that bioenergetic abnormalities precede treatment, representing disease features rather than iatrogenic artifacts. The animal model studies (Atkin et al., 2011; Steullet et al., 2010; Zhang et al., 2018; Xuan et al., 2015) enable causal mechanistic investigations impossible in human patients, while the cellular models (Park et al., 2015; Rosenfeld et al., 2011) permit controlled experimental manipulations to establish specific pathways from mitochondrial deficits to functional phenotypes.

However, important limitations temper interpretation and highlight priorities for future research. The substantial methodological heterogeneity across studies—varying patient populations (chronic vs. first-episode, medicated vs. naïve), tissue sources (specific brain regions, peripheral blood), assessment methods (enzyme assays, gene expression, respirometry, neuroimaging), and outcome measures—precluded quantitative meta-analysis. While this heterogeneity limits statistical synthesis, it provides complementary evidence across multiple analytical levels, from molecular mechanisms to systems-level brain function. Future studies employing standardized methodologies and outcome measures would enable meta-analytic quantification of effect sizes and exploration of moderating variables.

An important limitation of our review strategy concerns the exclusion of dopaminergic system terms from our search criteria. While this focused approach allowed detailed examination of mitochondrial-synaptic mechanisms, it may have limited identification of studies examining dopamine-mitochondria interactions or the role of bioenergetic dysfunction in dopaminergic pathway abnormalities. Given the well-established importance of dopaminergic dysfunction in schizophrenia, particularly for positive symptoms, and emerging evidence for bidirectional interactions between dopamine metabolism and mitochondrial function, future comprehensive reviews should explicitly incorporate neurotransmitter-system terms to capture the full complexity of these relationships.

The predominance of cross-sectional studies limits causal inference despite mechanistic insights from experimental models. Longitudinal studies tracking mitochondrial biomarkers and synaptic measures from prodrome through first episode and chronic illness would illuminate temporal dynamics: Does mitochondrial dysfunction precede psychosis onset? Does it progress with illness duration? Do specific trajectories predict outcomes? The study by Uranova et al. showing progression with illness duration provides suggestive evidence, but prospective longitudinal designs are needed to establish temporal relationships definitively. The regional specificity observed in *postmortem* studies deserves careful interpretation. While studies by Roberts et al. (2015), Karry et al. (2004), Sullivan et al. (2019a), consistently found prefrontal cortex most severely affected, *postmortem* interval, tissue preservation, and pH could differ across regions within individual brains. However, the consistency of regional patterns across multiple independent samples from different research groups using different methodologies argues for genuine biological heterogeneity rather than artifact. Future studies employing rapid autopsy protocols with systematic sampling across all brain regions would further clarify regional vulnerability patterns.

The translation from animal models to human pathology requires caution. While Atkin et al. (2011) DISC1-mutant mice exhibited mitochondrial, synaptic, and behavioral phenotypes consistent with schizophrenia, the translational validity of any single genetic model is limited. Schizophrenia likely results from polygenic risk interacting with environmental factors, and no single-gene model fully recapitulates the human disorder. Nevertheless, the consistency between rodent findings and human data—particularly the convergence on prefrontal cortex vulnerability and pyramidal neuron specificity—provides reassurance about translational relevance.

Critical gaps remain in our understanding. While we have documented what mitochondrial abnormalities exist, we know less about why they occur. Are they primarily genetic, reflecting inherited variants in nuclear or mitochondrial DNA? Are they environmental, resulting from prenatal infections, obstetric complications, or childhood stress? Most likely they reflect gene-environment interactions, with genetic vulnerabilities becoming manifest under environmental stress. Studies examining mitochondrial function in high-risk offspring of patients with schizophrenia could illuminate the relative contributions of genetic and environmental factors. The mechanistic links between specific mitochondrial deficits and particular synaptic phenotypes require further elucidation. While we know that complex I deficiency, ATP depletion, calcium dysregulation, and oxidative stress each contribute to synaptic dysfunction, the precise pathways and their relative contributions remain unclear. Does complex I deficiency cause synaptic dysfunction primarily through ATP depletion, or do impaired calcium buffering and oxidative stress play equal or greater roles? Answering these questions is not merely academic—it informs which therapeutic targets to prioritize.

## Conclusion

This systematic review provides compelling, converging evidence that mitochondrial bioenergetic impairment represents a core pathophysiological mechanism in schizophrenia, directly driving synaptic dysfunction that contributes to cognitive deficits and negative symptoms. Evidence spanning *postmortem* neurochemistry, *in vivo* neuroimaging, peripheral biomarkers, cellular models, and animal experiments establishes mitochondrial-synaptic dysfunction as a robust, clinically relevant, and potentially modifiable feature of the disorder.

Complex I and IV deficits contribute to ATP depletion, impaired calcium buffering, and oxidative stress that collectively impair synaptic transmission, plasticity, and cognitive function. Regional specificity—most pronounced in prefrontal cortex and hippocampus—and cell-type selectivity—affecting primarily pyramidal neurons and parvalbumin-positive interneurons—provide insights into why cognitive and negative symptoms are prominent and treatment-resistant. Bioenergetic abnormalities present at first episode, combined with progression over time, suggest critical intervention windows: early treatment could prevent progressive deterioration, while developmental intervention might prevent illness onset in high-risk individuals.

Clinically, this evidence justifies prioritizing mitochondrial function as a therapeutic target. Current treatments, while effective for positive symptoms, minimally address cognitive and motivational deficits impairing functional recovery. Mitochondrial-targeted interventions—pharmacological (NAC, coenzyme Q10, carnitine), dietary (ketogenic approaches), or cellular (mitophagy induction)—offer rational strategies for treatment-resistant symptoms. Peripheral biomarkers enabling patient stratification could enable precision medicine, identifying which patients benefit most from specific interventions.

Future research priorities include: (1) longitudinal studies tracking mitochondrial biomarkers from prodrome through illness course to establish temporal relationships and identify critical intervention windows; (2) mechanistic studies using causal manipulations combining genetic manipulation, mitochondrial assessment, electrophysiology, and behavioral testing to establish complete causal chains from mitochondrial deficits to phenotypes; (3) clinical trials of mitochondrial-targeted interventions, particularly in early illness when neuroprotection may be most effective, with rigorous designs assessing functional recovery and quality of life—combination therapies targeting multiple mitochondrial aspects simultaneously may prove superior to single-agent approaches; (4) biomarker validation in large samples with comprehensive clinical characterization to identify mitochondrial dysfunction subtypes benefiting from specific interventions; and (5) gene-environment interaction research examining how prenatal infections, obstetric complications, childhood stress, or substance use interact with genetic vulnerability to impair mitochondrial function, informing prevention strategies.

The evidence suggests we stand at a potential inflection point. For decades, the field focused almost exclusively on dopamine dysregulation, yielding medications effective for positive symptoms but leaving cognitive and negative symptoms largely untouched. By shifting focus to mitochondrial bioenergetics as fundamental pathophysiology, we open therapeutic avenues targeting biological substrates of treatment-resistant symptoms. The challenge is translating mechanistic understanding into interventions improving lives of patients and families affected by this devastating disorder.
