# Supplementary material for: Bioenergetic impairment in schizophrenia: role of mitochondrial signaling in synaptic dysfunction - a systematic review
Source: Front Cell Dev Biol. 2026 Feb 27;14:1740079. doi: 10.3389/fcell.2026.1740079 (PMC12982393; doi:10.3389/fcell.2026.1740079)
Supplement: Supplementary file 1 [file Table1.docx]

**Supplementary Table A) PRISMA Checklist for Systematic Review on Bioenergetic Impairment in Schizophrenia: Role of Mitochondrial Signaling in Synaptic Dysfunction**

| Section and Topic | Item # | Checklist item | Location where item is reported |
| --- | --- | --- | --- |
| TITLE |  |  |  |
| Title | 1 | Identify the report as a systematic review. | Page 1 |
| ABSTRACT |  |  |  |
| Abstract | 2 | See the PRISMA 2020 for Abstracts checklist. | Pages 1-2 |
| INTRODUCTION |  |  |  |
| Rationale | 3 | Describe the rationale for the review in the context of existing knowledge. | Pages 3-5 |
| Objectives | 4 | Provide an explicit statement of the objective(s) or question(s) the review addresses. | Page 5 |
| METHODS |  |  |  |
| Eligibility criteria | 5 | Specify the inclusion and exclusion criteria for the review and how studies were grouped for the syntheses. | Pages 6-7 |
| Information sources | 6 | Specify all databases, registers, websites, organizations, reference lists and other sources searched or consulted to identify studies. Specify the date when each source was last searched or consulted. | Page 6 |
| Search strategy | 7 | Present the full search strategies for all databases, registers and websites, including any filters and limits used. | Page 6 |
| Selection process | 8 | Specify the methods used to decide whether a study met the inclusion criteria of the review, including how many reviewers screened each record and each report retrieved, whether they worked independently, and if applicable, details of automation tools used in the process. | Page 7 |
| Data collection process | 9 | Specify the methods used to collect data from reports, including how many reviewers collected data from each report, whether they worked independently, any processes for obtaining or confirming data from study investigators, and if applicable, details of automation tools used in the process. | Page 7 |
| Data items | 10a | List and define all outcomes for which data were sought. Specify whether all results that were compatible with each outcome domain in each study were sought. | Page 7 |
|  | 10b | List and define all other variables for which data were sought. Describe any assumptions made about any missing or unclear information. | Page 7 |
| Study risk of bias assessment | 11 | Specify the methods used to assess risk of bias in the included studies, including details of the tool(s) used, how many reviewers assessed each study and whether they worked independently. | Pages 7-8 |
| Effect measures | 12 | Specify for each outcome the effect measure(s) used in the synthesis or presentation of results. | Page 8 |
| Synthesis methods | 13a | Describe the processes used to decide which studies were eligible for each synthesis. | Page 8 |
|  | 13b | Describe any methods required to prepare the data for presentation or synthesis, such as handling of missing summary statistics or data conversions. | Page 8 |
|  | 13c | Describe any methods used to tabulate or visually display results of individual studies and syntheses. | Page 8, Tables 1-7 |
|  | 13d | Describe any methods used to synthesize results and provide a rationale for the choice(s). If meta-analysis was performed, describe the model(s), method(s) to identify the presence and extent of statistical heterogeneity, and software package(s) used. | Page 8 |
|  | 13e | Describe any methods used to explore possible causes of heterogeneity among study results. | Page 8 |
|  | 13f | Describe any sensitivity analyses conducted to assess robustness of the synthesized results. | Not applicable |
| Reporting bias assessment | 14 | Describe any methods used to assess risk of bias due to missing results in a synthesis. | Page 8 |
| Certainty assessment | 15 | Describe any methods used to assess certainty in the body of evidence for an outcome. | Pages 7-8 |
| RESULTS |  |  |  |
| Study selection | 16a | Describe the results of the search and selection process, from the number of records identified in the search to the number of studies included in the review, ideally using a flow diagram. | Page 9, Figure 1 |
|  | 16b | Cite studies that might appear to meet the inclusion criteria, but which were excluded, and explain why they were excluded. | Page 9, Figure 1 |
| Study characteristics | 17 | Cite each included study and present its characteristics. | Pages 9-10, Table 1 |
| Risk of bias in studies | 18 | Present assessments of risk of bias for each included study. | Page 10, Table 4 |
| Results of individual studies | 19 | For all outcomes, present for each study: (a) summary statistics for each group and (b) an effect estimate and its precision, ideally using structured tables or plots. | Pages 10-20, Tables 1-2 |
| Results of syntheses | 20a | For each synthesis, briefly summarize the characteristics and risk of bias among contributing studies. | Pages 10-26 |
|  | 20b | Present results of all statistical syntheses conducted. If meta-analysis was done, present for each the summary estimate and its precision and measures of statistical heterogeneity. | Pages 10-26, Tables 2-5 |
|  | 20c | Present results of all investigations of possible causes of heterogeneity among study results. | Pages 27-29 |
|  | 20d | Present results of all sensitivity analyses conducted to assess the robustness of the synthesized results. | Not applicable |
| Reporting biases | 21 | Present assessments of risk of bias due to missing results for each synthesis assessed. | Pages 29-30 |
| Certainty of evidence | 22 | Present assessments of certainty in the body of evidence for each outcome assessed. | Pages 27-30 |
| DISCUSSION |  |  |  |
| Discussion | 23a | Provide a general interpretation of the results in the context of other evidence. | Pages 27-32 |
|  | 23b | Discuss any limitations of the evidence included in the review. | Pages 32-33 |
|  | 23c | Discuss any limitations of the review processes used. | Pages 32-33 |
|  | 23d | Discuss implications of the results for practice, policy, and future research. | Pages 30-32 |
| OTHER INFORMATION |  |  |  |
| Registration and protocol | 24a | Provide registration information for the review, including register name and registration number, or state that the review was not registered. | Page 6 |
|  | 24b | Indicate where the review protocol can be accessed, or state that a protocol was not prepared. | Page 6 |
|  | 24c | Describe and explain any amendments to information provided at registration or in the protocol. | Not applicable |
| Support | 25 | Describe sources of financial or other support for the review, and the role of the funders or sponsors in the review. | Page 35 |
| Competing interests | 26 | Declare any competing interests of review authors. | Page 35 |
| Availability of data, code and other materials | 27 | Report which of the following are publicly available and where they can be found: template data collection forms; data extracted from included studies; data used for all analyses; analytic code; any other materials used in the review. | Page 35 |

**Supplementary Table B) Methodological Quality Assessment Using the Newcastle-Ottawa Scale Quality assessment of included studies using the Newcastle-Ottawa Scale (NOS) for observational studies**

| Study | Selection (0-4) | Comparability (0-2) | Outcome/Exposure (0-3) | Total NOS Score (/9) | Risk of Bias |
| --- | --- | --- | --- | --- | --- |
| Roberts et al. | 4 | 2 | 2 | 8 | Low |
| Maurer et al. | 3 | 2 | 2 | 7 | Low |
| Sullivan et al. (Study 1) | 4 | 2 | 2 | 8 | Low |
| Karry et al. (2004) | 3 | 2 | 2 | 7 | Low |
| Yao et al. | 3 | 2 | 2 | 7 | Low |
| Uranova et al. (2023) | 3 | 2 | 2 | 7 | Low |
| Du et al. | 4 | 2 | 2 | 8 | Low |
| Yuksel et al. | 4 | 2 | 3 | 9 | Very Low |
| Rowland et al. | 4 | 2 | 2 | 8 | Low |
| Ben-Shachar et al. (2007) | 4 | 2 | 2 | 8 | Low |
| Do et al. | 4 | 2 | 2 | 8 | Low |
| Sullivan et al. (Study 2) | 3 | 2 | 2 | 7 | Low |
| Akarsu et al. (2015) | 3 | 2 | 2 | 7 | Low |
| Bergman et al. (2019) | 3 | 2 | 2 | 7 | Low |
| Xia et al. (2021) | 4 | 2 | 2 | 8 | Low |
| Scaini et al. (2018) | 3 | 2 | 2 | 7 | Low |
| Chen et al. (2023) | 3 | 2 | 1 | 6 | Medium |
| Park et al. | 4 | 2 | 2 | 8 | Low |
| Robicsek et al. (2013) | **3** | **2** | **2** | **7** | **Low** |
| Norkett et al. | 3 | 2 | 2 | 7 | Low |
| Ni et al. | 3 | 2 | 2 | 7 | Low |
| Ben-Shachar et al. (2004) | 3 | 2 | 2 | 7 | Low |
| Steullet et al. | 4 | 2 | 2 | 8 | Low |
| Dror et al. | 3 | 2 | 2 | 7 | Low |
| Rosenfeld et al. (2011) | 4 | 2 | 2 | 8 | Low |
| Garcia-de la Cruz et al. (2024) | 3 | 2 | 2 | 7 | Low |
| Ben-Shachar et al. (2015) | 2 | 2 | 2 | 6 | Medium |
| Bar-Yosef et al. (2020) | 4 | 2 | 2 | 8 | Low |
| Atkin et al. | 4 | 2 | 2 | 8 | Low |
|  |  |  | **Summary Statistics:** |  |  |
|  |  |  | **Median Score:** | **7** |  |
|  |  |  | **Range:** | **6-9** |  |
|  |  |  | **High Quality (≥7):** | **26 studies (90%)** |  |
|  |  |  | **Medium Quality (5-6):** | **3 studies (10%)** |  |

**Supplementary Table C: Variables Extracted from Studies for Systematic Review on Bioenergetic Impairment in Schizophrenia: Role of Mitochondrial Signaling in Synaptic Dysfunction**

| All Studies | Postmortem/Neuroimaging Studies | Longitudinal/Mechanistic Studies |
| --- | --- | --- |
| Study Identification | **Tissue/Imaging Details** | **Temporal Measurements** |
| Author, Year | Brain Region Analyzed | Follow-up Duration |
| Country/Region | Tissue Quality (PMI, pH) | Assessment Time Points |
| Study Design | **Imaging Modality (MRS, PET)** | **Baseline Characteristics** |
| Sample Size (SCZ/Controls) | Scanner Parameters | Age at Follow-up |
| Publication Language | Voxel Placement | Disease Progression Patterns |
|  | Regional Specificity | State Changes (Acute/Residual) |
|  |  |  |
| Participant Characteristics | **Methodological Approach** | **Conversion Outcomes** |
| Age Range (mean, SD) | Postmortem Analysis | Conversion to Chronicity |
| Gender Distribution | ³¹P-MRS Spectroscopy | Time to Conversion |
| Ethnicity/Race | FDG-PET Imaging | Functional Trajectories |
| Socioeconomic Status | Cell Culture Studies | Academic Performance |
| Geographic Location | iPSC-derived Neurons | Occupational Outcomes |
|  | Animal Models |  |
|  | Lymphoblastoid Lines | Predictive Validity |
| Diagnosis and Clinical State | **Western Blot Analysis** | **Symptom Prediction** |
| Diagnostic Criteria (DSM/ICD) | qPCR/RNA-seq | Sensitivity/Specificity |
| First-Episode vs Chronic | Immunohistochemistry | Positive/Negative Predictive Value |
| Drug-Naïve vs Treated | Electron Microscopy | Biomarker Performance |
| Duration of Illness |  |  |
| Age of Onset | Cell-Type Specificity | Statistical Analyses |
| Symptom Severity (PANSS) | Pyramidal Neurons | Effect Size Calculations |
| BPRS, SAPS, SANS Scores | Interneurons (PV, SST) | Percentage Change |
| Comorbidities | Astrocytes | Cohen's d Values |
| Substance Use | Microglia | Correlation Coefficients |
| Trauma History | Oligodendrocytes | Regression Models |
|  |  | Survival Analysis |
| Mitochondrial Assessment | **Synaptic Markers** | **Missing Data Handling** |
| Complex I Activity | Synaptic Density |  |
| Complex II Activity | PSD-95 Expression | Quality Assessment |
| Complex III Activity | Synapsin Expression | Newcastle-Ottawa Scale |
| Complex IV Activity | Spinophilin Levels | Selection Bias |
| Complex V Activity | Dendritic Spine Density | Comparability |
| Electron Transport Chain | Synaptosomal Fractions | Outcome Assessment |
|  |  | Attrition Analysis |
| Bioenergetic Parameters | **Mitochondrial Ultrastructure** |  |
| ATP Synthesis Rate | Mitochondrial Density | Developmental Considerations |
| ATP/ADP Ratio | Mitochondrial Size | Age-Appropriate Measures |
| Phosphocreatine (PCr) | Cristae Structure | Developmental Trajectories |
| Creatine Kinase Flux | Mitochondrial Distribution | Maturational Effects |
| Pi/ATP Ratio | Axonal vs Dendritic | Critical Periods |
| NAD+/NADH Ratio | Synaptic vs Soma | Neurodevelopmental Windows |
|  |  |  |
| Mitochondrial Structure/Dynamics | **Genetic/Molecular Analysis** | **Intervention Implications** |
| Membrane Potential (Δψm) | Gene Expression (mRNA) | Treatment Response |
| JC-1 Dye Assessment | Protein Expression (Western) | Medication Effects |
| Mitochondrial Network | **mtDNA Copy Number** | **Antipsychotic Impact** |
| Fusion Proteins (Mfn1/2, OPA1) | mtDNA Mutations | Clozapine Effects |
| Fission Proteins (Drp1, Fis1) | Nuclear-Encoded Genes | Olanzapine Effects |
| Mitochondrial Transport | **NDUFV1, NDUFV2** | **Rescue Experiments** |
| Kinesin/Dynein Motors | NDUFV2P1 Pseudogene | NAC/GSH Treatment |
| Axonal Transport Velocity | CPEB1 Expression | CoQ10 Supplementation |
|  | DISC1 Mutations | Urolithin A Effects |
| Oxidative Stress | **22q11.2 Deletions** | **Cognitive Remediation** |
| GSH (Glutathione) Levels |  |  |
| GSH/GSSG Ratio | Pharmacological Studies | Clinical Translation |
| Lipid Peroxidation | Dopamine Toxicity | Screening Protocols |
| 4-HNE Levels | Complex I Inhibitors | Implementation Barriers |
| MDA (Malondialdehyde) | Mitochondrial Toxins | Training Requirements |
| Protein Carbonylation | Neuroprotective Agents | Cost-Effectiveness |
| 8-oxo-dG (DNA Damage) | Dose-Response | Healthcare Integration |
| ROS Generation | Rescue Mechanisms |  |
| Antioxidant Enzymes |  | Methodological Quality |
| SOD (Superoxide Dismutase) | Functional Consequences | Study Design Quality |
| Catalase | Synaptic Transmission | Sample Representativeness |
| Glutathione Peroxidase | Release Probability | Follow-up Completeness |
|  | Paired-Pulse Facilitation | Blinding Procedures |
| Calcium Homeostasis | **Miniature EPSCs/IPSCs** | **Inter-rater Reliability** |
| Intracellular [Ca²⁺] | LTP/LTD Induction | Practice Effects Control |
| Mitochondrial Ca²⁺ Uptake | **Action Potentials** |  |
| MCU Expression | Neuronal Excitability | Bias Assessment |
| ER-Mitochondria Contacts (MAMs) | Network Oscillations | Confounding Variables |
| IP3R, VDAC, Grp75 | Gamma Oscillations | Medication Confounds |
| Ca²⁺ Transient Amplitude |  | Substance Use |
| Ca²⁺ Decay Kinetics | Behavioral Phenotypes | Smoking Status |
| Buffering Capacity | Prepulse Inhibition (PPI) | Educational Level |
|  | T-maze Performance | Socioeconomic Status |
| Clinical Correlations | **Social Interaction** | **PMI (Postmortem)** |
| Symptom Domains | Nest Building | Tissue Quality |
| Positive Symptoms | Locomotor Activity |  |
| Negative Symptoms |  | Study Limitations |
| Cognitive Deficits | Regional Heterogeneity | Sample Size |
| Executive Function | Prefrontal Cortex | Methodological Constraints |
| Working Memory | Hippocampus | Generalizability |
| Verbal Memory | Anterior Cingulate | Temporal Resolution |
| Processing Speed | Temporal Cortex | Cross-sectional vs Longitudinal |
| Attention/Vigilance | Basal Ganglia | Causality Determination |
| Social Cognition | Parietooccipital Cortex |  |
|  |  | Funding and Support |
| Network Connectivity | **Data Quality** | **Funding Sources** |
| Default Mode Network | **Missing Data Rates** | **Conflicts of Interest** |
| Task-Positive Networks | **Attrition Patterns** | **Industry Involvement** |
| DMN-TPN Anticorrelation | Measurement Reliability | Author Affiliations |
| Functional Connectivity | Validity Evidence | Grant Numbers |
| Graph Theory Metrics | Replication Attempts | Institutional Support |
|  |  |  |
| Main Outcomes | **Effect Estimates** | **Key Findings** |
| Primary Outcomes | Quantitative Data | Mitochondrial Dysfunction Pattern |
| Secondary Outcomes | Mean Differences | Synaptic Impairment Pattern |
| Tertiary Outcomes | Standard Deviations | Regional Vulnerability |
| Functional Outcomes | Effect Sizes (d, r) | Mechanistic Pathways |
| Academic Performance | Confidence Intervals | Clinical Relationships |
| Social Functioning | P-values | Therapeutic Targets |
| Quality of Life | **Statistical Power** | **Biomarker Potential** |

**Note:** *Variables listed in the "All Studies" column were extracted for all included studies. Additional variables in the second column were extracted when applicable to postmortem tissue analyses or neuroimaging investigations. Variables in the third column were extracted for longitudinal studies examining disease progression, treatment response, and mechanistic relationships. This comprehensive extraction framework enabled systematic comparison across diverse methodological approaches while capturing study-specific details essential for interpreting mitochondrial-synaptic dysfunction in schizophrenia and its clinical implications.*
